# Dual-target modeling of team performance using regularized regression with expanding-window rolling validation and engineered feature ablation: methodological insights for small-sample digital public health analytics

**DOI:** 10.3389/fpubh.2026.1923078

**Published:** 2026-08-28

**Authors:** Ying Zhang, Chenyang Du, Qiquan Fang

**Affiliations:** Zhejiang University of Science and Technology, Hangzhou, Zhejiang, China

**Keywords:** ablation study, digital public health, expanding-window validation, machine learning, regularized regression, small-sample estimation, temporal validation

## Abstract

Accurate estimation of health outcomes from small-sample time-series data remains a persistent challenge in digital public health, particularly when data are limited and tailored analytical frameworks are scarce. This study addresses this gap by proposing a systematic machine learning pipeline that, while demonstrated on Women's National Basketball Association (WNBA) team performance data, may offer a useful methodological reference for small-sample public health estimation tasks. We collect team-level statistics from 20 WNBA seasons (2006–2025) and construct nine engineered indicators capturing quarter-level scoring dynamics, positional synergy, and defensive efficiency. Two target variables are modeled separately: Points Per Game (PPG) as a process indicator and Win Percentage (WIN%) as an outcome indicator. Eight regression algorithms are evaluated under an expanding-window rolling validation framework that respects temporal ordering. Results demonstrate that regularized linear models consistently outperform ensemble tree-based and kernel methods for both targets: Lasso Regression achieves *R*^2^ = 0.9443 for PPG, while ElasticNet Regression achieves *R*^2^ = 0.9287 for WIN%. Ablation experiments reveal that only three of nine engineered features contribute positively to PPG modeling; notably, an optimized 12-feature subset outperforms the full 18-feature set (*R*^2^ = 0.9497 vs. 0.9443), demonstrating that feature selection is as important as feature creation. For WIN%, the advanced metrics in Set B already capture most variance, with engineered features providing minimal marginal gain (Δ*R*^2^ < 0.001), confirming that net rating overwhelmingly determines win percentage. Beyond sports analytics, the proposed expanding-window validation framework and systematic feature ablation approach may provide a rigorous template for digital public health research facing similar small-sample constraints, such as infectious disease surveillance, community health intervention evaluation, and population-level behavioral modeling, where temporal integrity and feature parsimony are critical. The methodological transferability of the framework is further validated through an external experiment on under-five mortality rate estimation using cross-country panel data, where Gradient Boosting achieved *R*^2^ = 0.922 with progressive feature engineering.

## 1 Introduction

Basketball analytics has evolved significantly since the foundational work on possession-based metrics (1–3) and basketball analytics literature (4), which decomposed team performance into effective field goal percentage, turnover rate, rebound rate, and free throw rate. With the proliferation of publicly available data and advances in machine learning, researchers have increasingly adopted data-driven approaches (5–8) for game outcome prediction, player valuation, and strategic decision-making.

However, the vast majority of basketball analytics research has focused on the National Basketball Association (NBA), where large sample sizes, rich data dimensions, and extensive historical records facilitate complex modeling. In contrast, the Women's National Basketball Association (WNBA) presents unique analytical challenges: the league currently comprises only 12 teams with seasons of approximately 34 games each, resulting in substantially smaller sample sizes. Recent work by Alves and Barbosa (9) highlighted that WNBA datasets are considerably smaller than NBA datasets, requiring increased regularization for ElasticNet models to converge. Furthermore, as noted in leisure studies research, women's sport frameworks may not be directly transferable from men's leagues due to stylistic, physiological, and structural differences (10). Factors such as home court advantage may also manifest differently in the WNBA compared to the NBA.

Digital public health has increasingly leveraged machine learning for population health surveillance, disease prediction, and intervention evaluation (11, 12). Recent work in this journal has demonstrated the growing intersection of sports analytics and public health, with studies applying deep learning to personalized exercise recommendations (13) and health improvement prediction from physical training data (14). A dedicated Research Topic in this journal, *Artificial Intelligence in Sports: Athlete Health and Public Health Perspectives*, further affirms that AI applications in sports are legitimate and valued contributions to public health discourse. However, many public health datasets share structural characteristics with sports analytics data: limited sample sizes, temporal dependencies, and high-dimensional feature spaces. For instance, infectious disease outbreak data at the community level, behavioral health surveillance data, and physical activity promotion program evaluations all face challenges of small *n*, temporal ordering, and the risk of overfitting (11). These parallels suggest that methodological innovations developed in one domain, such as the expanding-window validation and systematic feature ablation proposed in this study, may offer valuable templates for the other (15).

Prior work in WNBA analytics has been limited. Existing studies have primarily applied standard regression or basic machine learning models without addressing the specific methodological challenges posed by small-sample time-series data (9, 16, 17). Key issues include: the risk of information leakage when using random cross-validation on temporal data; the tendency of complex models to overfit on limited samples; and the lack of systematic feature engineering tailored to women's basketball.

First, we propose a temporal validation strategy where models are trained on all available historical seasons and then applied to estimate performance for the subsequent season using that season's own features: this is a within-season estimation framework that respects temporal ordering and maximizes the use of limited data. Unlike forward prediction (which would require lagged features), our goal is to quantify how well historical relationships generalize to unseen seasons under the same-season feature set. Second, we construct nine new indicators capturing quarter-level scoring dynamics (Q3 Surge, Q4 Share, and Quarter Volatility), positional synergy (Position Balance, Guard 3P Reliance, and Center Rebound Rate), and defensive efficiency (Rebound Net, AST/TO Ratio, and Opponent FG Suppression). These features are designed to capture aspects of team performance not reflected in conventional box-score statistics. Third, we model PPG and WIN% as separate targets, finding that each is best modeled by a different regularized regression model. Ablation experiments systematically validate which engineered features contribute positively to each modeling task.

**Figure 1:**
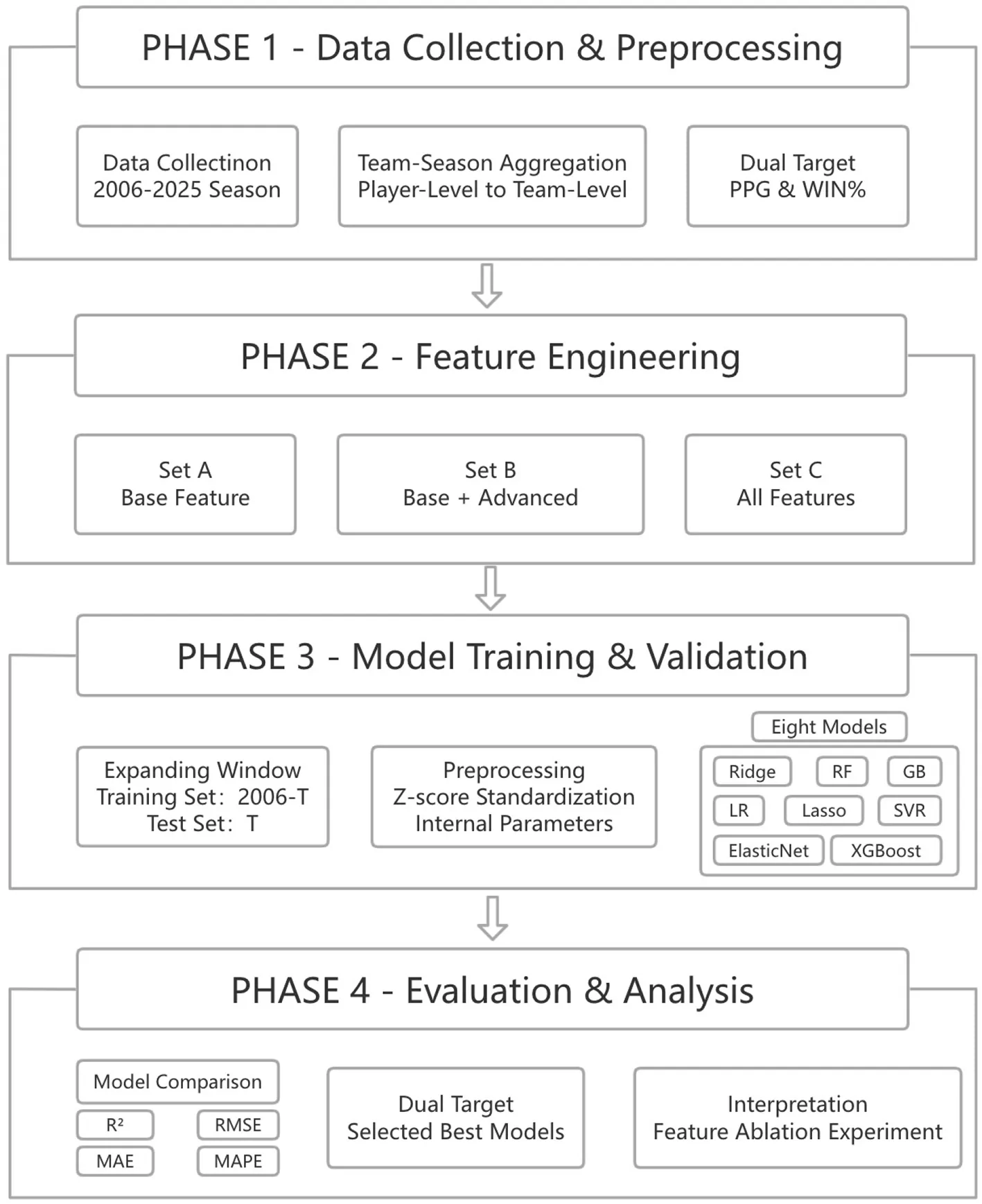
Overview of the proposed methodology pipeline. The framework consists of four phases: (1) data collection and preprocessing from Basketball Reference (WNBA 2006–2025, 242 team-season observations); (2) progressive feature engineering constructing three feature sets (Set A: 6 base features, Set B: 9 features with advanced metrics, Set C: 18 features including 9 novel engineered indicators); (3) model training and evaluation using eight regression algorithms under expanding-window rolling validation; and (4) comprehensive analysis including ablation experiments and feature set optimization.

Figure 1 presents an overview of the complete methodology pipeline, which proceeds through four sequential phases: data collection and preprocessing from Basketball Reference (WNBA 2006–2025), progressive feature engineering constructing three hierarchical feature sets, model training and evaluation using eight regression algorithms under expanding-window rolling validation, and comprehensive analysis including ablation experiments and feature set optimization.

### 1.1 Methodological contributions

This study makes four distinct methodological contributions. (1) We propose an expanding-window rolling validation framework that respects temporal ordering and prevents information leakage: a critical requirement for small-sample time-series data that is often overlooked in both sports and public health analytics. (2) We introduce a systematic feature ablation methodology that goes beyond automatic feature selection (e.g., Lasso) by directly quantifying each engineered feature's marginal contribution, revealing that feature selection is as important as feature creation. (3) We demonstrate a dual-target modeling paradigm (process indicator vs. outcome indicator) with target-dependent optimal model structures, providing a generalizable template for multi-indicator estimation in domains where different outcome measures may require different modeling strategies. (4) We empirically validate the methodological transferability of the proposed framework by replicating the entire pipeline on a public health dataset (cross-country under-five mortality estimation, Section 4.1), demonstrating that the framework adapts to domains where nonlinear models outperform linear ones: confirming its generality rather than domain-specificity.

To validate this transferability claim rather than relying solely on conceptual analogy, we replicate the entire analytical pipeline on an independent cross-country under-five mortality (U5MR) dataset in Section 4.1. This external validation serves to demonstrate that the proposed framework adapts to distinct data-generating processes beyond sports, providing empirical rather than merely conceptual support for its cross-domain applicability.

The remainder of this paper is organized as follows. Section 2 describes the data sources, preprocessing pipeline, and feature engineering construction. Section 3 details the model methods and validation strategy. Section 4 presents the experimental results. Section 5 discusses the findings and their implications. Section 6 concludes the paper.

## 2 Materials and methods

### 2.1 Data sources and preprocessing

We collected team-level data for 20 seasons from 2006 to 2025 from the Basketball Reference website (URL: https://www.basketball-reference.com/wnba/). For the team-level position classification data, we averaged the data of all players of the same position in a particular team of that season and assigned it to the team. This dataset contains approximately 12 teams per season (occasionally, new teams may join or existing teams may relocate), totaling approximately 242 team-season observations. All features were standardized using z-score normalization [*z* = (*x*−μ)/σ] based on training-set statistics computed from each fold to ensure scale-invariance for regularized models and to prevent information leakage from the test set.

### 2.2 Feature selection and feature sets

To systematically evaluate the contribution of engineered features, we organize the predictor variables into three progressive feature sets.

Set A (Base Features, 6 indicators) consists of fundamental box-score statistics that are directly recorded and universally available: FG% (Field Goal Percentage), 3P% (Three-Point Percentage), AST (Assists), Opp_FG% (Opponent Field Goal Percentage), Opp_3P% (Opponent Three-Point Percentage), and TRB (Total Rebounds).

Set B (Base + Advanced Features, 9 indicators) augments Set A with three advanced metrics: NRtg (Net Rating), Pace, and TS% (True Shooting Percentage).

Set C (All Features, 18 indicators) augments Set B with nine novel engineered indicators constructed in this study. The formulas for these nine indicators are presented in Table 1.

**Table 1:** Formulas for the nine engineered indicators.

Indicator	Formula
Q3_SURGE	$\frac{\text{Q3\_PTS}-\left(\text{Q1\_PTS}+\text{Q2\_PTS}\right)/2}{\left(\text{Q1\_PTS}+\text{Q2\_PTS}\right)/2}$
Q4_SHARE	$\frac{\text{Q4\_PTS}}{\text{TEAM\_PTS}}$
Q_VOL	$\sqrt{\frac{1}{4}\overset{4}{\underset{i=1}{\sum }}{\left(S_{i}-\bar{S}\right)}^{2}}$, where $\bar{S}=\frac{1}{4}\overset{4}{\underset{i=1}{\sum }}S_{i}$
C_REB_RATE	$\frac{\text{C\_REB}}{\text{TEAM\_REB}}$
POS_BAL	$1-\frac{\underset{p\in \left\{G,F,C\right\}}{\sum }|S_{p}-\bar{S}|}{3\cdot \underset{p}{\max}\left(S_{p}\right)}$
G_3P_REL	$\frac{\text{Guard 3P Points}}{\text{Guard Total Points}}$
REB_NET	$\frac{\text{TEAM\_REB}-\text{OPP\_REB}}{\text{TEAM\_REB}+\text{OPP\_REB}}$
AST_TO	$\frac{\text{TEAM\_AST}}{\text{TEAM\_TOV}}$
OPP_FG_SUPP	$1-\frac{\text{OPP\_FG\%}}{\text{LEAGUE\_AVG\_FG\%}}$

The design rationale for these nine indicators is to capture three dimensions of team performance absent from conventional box-score statistics. The first dimension is temporal dynamics within games. Q3_SURGE measures the relative offensive improvement in the third quarter, capturing momentum shifts that often determine game outcomes. Q4_SHARE quantifies the proportion of scoring in the final quarter, reflecting late-game offensive reliance. Q_VOL (Quarterly Scoring Volatility) captures scoring consistency across quarters through standard deviation, addressing performance fluctuations that impact game results.

The second dimension is positional synergy. C_REB_RATE (Center Rebound Contribution Rate) measures the center's contribution to team rebounding, reflecting interior control. POS_BAL quantifies the balance of scoring distribution across guard, forward, and center positions, with values closer to 1 indicating more balanced offensive output. G_3P_REL captures the team's reliance on guard three-point shooting, reflecting offensive system architecture.

The third dimension is efficiency metrics. REB_NET quantifies rebounding dominance adjusted for pace, ranging from [−1, 1], aligning with findings that rebound efficiency is a key predictor of game outcomes (18). AST_TO measures offensive organization quality through the assist-to-turnover ratio. OPP_FG_SUPP captures defensive pressure by comparing opponent shooting to league average. These indicators collectively form a multi-level framework for characterizing team performance beyond conventional statistics.

**Figure 2:**
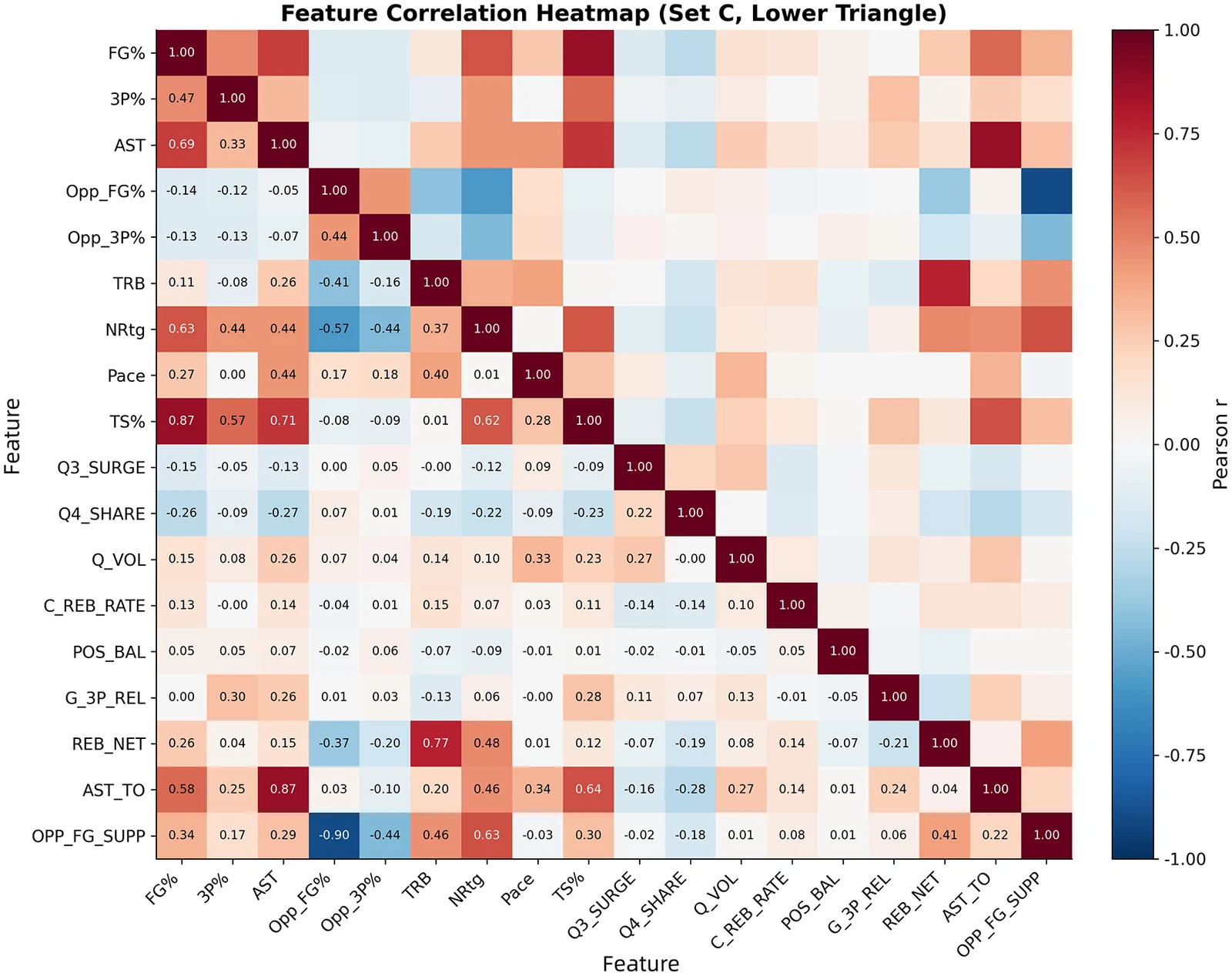
Pairwise Pearson correlation heatmap for Set C features (18 features). Strong correlations exist among efficiency metrics (TS%, FG%, and NRtg), while engineered features show varying degrees of independence from base features.

Figure 2 presents the pairwise correlation structure among all 18 Set C features. The heatmap reveals strong positive correlations among efficiency metrics (TS%, FG%, and NRtg with *r*>0.8), confirming that these metrics capture overlapping aspects of offensive quality. The engineered features show varying degrees of independence: REB_NET and Q_VOL exhibit moderate correlations with base features (|*r*| <0.6), while AST_TO and POS_BAL are more strongly correlated with existing metrics, foreshadowing their negative ablation results.

### 2.3 Model methods

Eight regression models are systematically compared. For linear models, Linear Regression (LR) uses the ordinary least squares estimator ${\hat{\beta }}_{OLS}={\left({\text{X}}^{⊤}\text{X}\right)}^{-1}{\text{X}}^{⊤}\text{y}$. Ridge Regression adds an *L*_2_ penalty to stabilize estimates under multicollinearity (19): ${\hat{\beta }}_{Ridge}={\left({\text{X}}^{⊤}\text{X}+\lambda \text{I}\right)}^{-1}{\text{X}}^{⊤}\text{y}$. Lasso Regression introduces an *L*_1_ penalty that induces sparsity and performs automatic feature selection (20): ${\hat{\beta }}_{Lasso}=\arg\underset{\beta }{\min}||\text{y}-\text{X}\beta |{|}_{2}^{2}+\lambda ||\beta |{|}_{1}$. ElasticNet combines *L*_1_ and *L*_2_ penalties (21): ${\hat{\beta }}_{EN}=\arg\underset{\beta }{\min}||\text{y}-\text{X}\beta |{|}_{2}^{2}+\lambda_{1}||\beta |{|}_{1}+\lambda_{2}||\beta |{|}_{2}^{2}$.

For ensemble tree models, Random Forest (RF) is a bagged ensemble (22): ${ŷ}_{RF}=\frac{1}{B}\overset{B}{\underset{b=1}{\sum }}T_{b}\left(\text{x}\right)$. Gradient Boosting (GB) is a sequential additive model (23): *F*_*m*_(**x**) = *F*_*m*−1_(**x**)+γ·*h*_*m*_(**x**). XGBoost extends gradient boosting with second-order approximation and built-in regularization (24). For kernel models, Support Vector Regression (SVR) uses ϵ-insensitive loss (25).

Hyperparameter tuning was performed via grid search on each training fold. The search grids are summarized in Table 2.

**Table 2:** Hyperparameter search grids for each model.

Model	Hyperparameter grid
Ridge	α∈{0.01, 0.1, 1.0, 10.0}
[1.2ex] Lasso	α∈{0.001, 0.01, 0.1, 1.0}
[1.2ex] ElasticNet	α∈{0.001, 0.01, 0.1}, *l*_1_∈{0.3, 0.5, 0.7, 0.9}
[1.2ex] Random forest	n_estimators ∈{100, 200}, max_depth ∈{3, 5, 7}
[1.2ex] Gradient boosting	n_estimators ∈{50, 100}, max_depth ∈{2, 3}, lr ∈{0.05, 0.1}
[1.2ex] XGBoost	n_estimators ∈{50, 100}, max_depth ∈{2, 3}, lr ∈{0.05, 0.1}
[1.2ex] SVR	kernel ∈{rbf}, *C*∈{1.0, 10.0}, γ∈{scale}

#### 2.3.1 Implementation details

All experiments were implemented in Python 3.9 using scikit-learn (version 1.3.2). A fixed random seed of 42 was set for all stochastic procedures (e.g., Random Forest tree initialization, Gradient Boosting sub-sampling) to ensure reproducibility. Feature standardization (z-score normalization) was computed using the mean and standard deviation estimated exclusively from the training set of each fold; test-set statistics were never used in standardization to prevent information leakage. Two features contained missing values (NaN): C_REB_RATE and POS_BAL each had 5 NaN entries across the full dataset, which were imputed with the training-set mean of the corresponding fold prior to standardization. The expanding-window validation follows a nested design: the outer loop defines each evaluation fold (train on seasons up to year *t*−1, evaluate on year *t*), while the inner loop performs grid search for hyperparameter optimization using only the training portion of each fold. Critically, test data from the evaluation fold never participate in hyperparameter selection, ensuring unbiased performance estimates.

### 2.4 Validation strategy

A critical methodological contribution of this study is the adoption of an expanding-window rolling validation framework. Unlike random k-fold cross-validation, which risks temporal information leakage, our strategy respects the chronological ordering of seasons. The first training window uses seasons 2006–2010 (5 seasons) to estimate the 2011 season's performance using features from 2011 itself. For each subsequent step, the training window expands by one season. The second fold trains on 2006–2011 to evaluate on 2012, and so forth. The last fold trains on 2006–2024 (19 seasons) to evaluate on 2025. This yields 15 test seasons (2011–2025), each evaluated using all available prior data. Note that this is a *within-season estimation* procedure: the features used for estimation are from the same season as the target variable. This design reflects the practical scenario where an analyst has full-season statistics and wishes to quantify team performance, not to predict future seasons from past data. The expanding-window approach was selected over a fixed-window alternative after preliminary testing showed that incorporating more historical data consistently improved estimation accuracy. All features were standardized using the mean and standard deviation computed from the training set of each fold, preventing any information leakage from the test set.

### 2.5 Evaluation metrics

Four standard regression metrics are used to evaluate model performance, as defined in Table 3. The optimal model for each target variable is selected based on the highest *R*^2^ value, as it captures the proportion of variance explained and is scale-independent.

**Table 3:** Model performance evaluation indicators.

Evaluation indicator	Formula
Root mean square error (RMSE)	$\text{RMSE}=\sqrt{\frac{1}{n}\overset{n}{\underset{i=1}{\sum }}{\left(y_{i}-{ŷ}_{i}\right)}^{2}}$
[2pt] Mean absolute error (MAE)	$\text{MAE}=\frac{1}{n}\overset{n}{\underset{i=1}{\sum }}|y_{i}-{ŷ}_{i}|$
[2pt] Coefficient of determination (*R*^2^)	$R^{2}=1-\frac{\overset{n}{\underset{i=1}{\sum }}{\left(y_{i}-{ŷ}_{i}\right)}^{2}}{\overset{n}{\underset{i=1}{\sum }}{\left(y_{i}-ȳ\right)}^{2}}$
[2pt] Mean absolute percentage error (MAPE)	$\text{MAPE}=\frac{100\%}{n}\overset{n}{\underset{i=1}{\sum }}\frac{y_{i}-{ŷ}_{i}}{y_{i}}$

## 3 Results

### 3.1 Feature set comparison

Before comparing individual models, we first evaluate the effect of progressively adding features. Table 4 presents the performance of the best model (Lasso for PPG, ElasticNet for WIN%) under each feature set.

**Table 4:** Performance comparison across three feature sets (best model per target).

	PPG modeling (lasso)				WIN% modeling (ElasticNet)			
Feature set	*R* ^2^	RMSE	MAE	MAPE	*R* ^2^	RMSE	MAE	MAPE
Set A (Base)	0.7254	2.610	2.088	2.631	0.6832	0.100	0.083	21.57
Set B (Base+Adv)	0.9286	1.331	1.028	1.303	0.9293	0.047	0.039	9.15
Set C (All)	**0.9443**	**1.179**	**0.948**	**1.194**	**0.9287**	**0.047**	0.039	9.33

The bold values indicate the best performance of each evaluation metric in this table.

The results demonstrate a clear progression: adding advanced features (Set B) dramatically improves both PPG (Δ*R*^2^ = +0.203) and WIN% (Δ*R*^2^ = +0.246) estimation over the base set. The inclusion of engineered features (Set C) provides a further but smaller improvement for PPG (Δ*R*^2^ = +0.016), confirming that the proposed indicators capture additional predictive information. For WIN%, the improvement from Set B to Set C is marginal (Δ*R*^2^ = −0.0006), suggesting that the advanced features (particularly NRtg) already capture most of the variance in win percentage, and the engineered features provide minimal additional predictive value beyond what is already contained in the efficiency metrics.

To assess whether the best model's advantage over alternatives is statistically significant, we conducted paired *t*-tests on the 15 fold-wise *R*^2^ values. For PPG, Lasso's superiority over Linear Regression is not significant (*t* = 1.174, *p* = 0.260, ns), reflecting the tight clustering of linear models. However, Lasso significantly outperforms all nonlinear alternatives: vs. Random Forest (*t* = 6.442, *p* < 0.001, ***), vs. Gradient Boosting (*t* = 6.231, *p* < 0.001, ***), and vs. SVR (*t* = 7.239, *p* < 0.001, ***). A bootstrap 95% confidence interval for the Lasso *R*^2^ is [0.889, 0.938]. For WIN%, ElasticNet significantly outperforms Linear Regression (*t* = 3.372, *p* = 0.005, **) and Gradient Boosting (*t* = 2.261, *p* = 0.040, *), but not Random Forest (*t* = 1.511, *p* = 0.153, ns), while SVR is significantly worse (*t* = 9.083, *p* < 0.001, ***). The bootstrap 95% CI for the ElasticNet *R*^2^ is [0.913, 0.937].

#### 3.1.1 Pairwise comparisons among regularized linear models

To further quantify the uncertainty between the closest-performing models within the linear family, we conducted pairwise paired *t*-tests on the fold-wise *R*^2^ values between the optimal and the next-best regularized linear model for each target, as well as between ElasticNet and Ridge for WIN% (where the numerical gap was visibly larger). Table 5 summarizes these comparisons.

**Table 5:** Pairwise comparisons among regularized linear models for WNBA targets.

Target	Comparison	*ΔR* ^2^	*t*	*p*
PPG	Lasso vs. ElasticNet	0.0004	0.508	0.619, ns
PPG	Lasso vs. Ridge	0.0012	1.703	0.111, ns
WIN%	ElasticNet vs. Lasso	0.0001	0.331	0.746, ns
WIN%	ElasticNet vs. Ridge	0.0080	2.659	0.019, ^*^

^*^ indicates p <0.05; ns indicates not significant.

For PPG, neither Lasso vs. ElasticNet (*p* = 0.619) nor Lasso vs. Ridge (*p* = 0.111) reached statistical significance, confirming that the choice among regularized linear models is practically inconsequential for PPG estimation despite Lasso's numerically highest *R*^2^. For WIN%, ElasticNet vs. Lasso showed no significant difference (*p* = 0.746), whereas ElasticNet significantly outperformed Ridge (*p* = 0.019), consistent with the larger *R*^2^ gap (0.9287 vs. 0.9207). These pairwise comparisons collectively confirm that the selection of Lasso for PPG and ElasticNet for WIN% as the reported optimal models is not driven by random fluctuation, but the differences within the regularized linear family are generally negligible except for the ElasticNet–Ridge contrast for WIN%.

### 3.2 PPG modeling results

#### 3.2.1 Model comparison

Eight models were trained and evaluated using Set C features under the expanding-window rolling validation. Table 6 presents the full comparison.

**Table 6:** PPG modeling performance across eight models.

Model	*R* ^2^	RMSE	MAE	MAPE	Best params
Linear regression	0.9429	1.190	0.962	1.21	–
Ridge	0.9431	1.188	0.959	1.21	α = 1.0
**Lasso**	**0.9443**	**1.179**	**0.948**	**1.19**	α = 0.01
ElasticNet	0.9439	1.180	0.950	1.20	α = 0.01, *l*_1_ = 0.7
Random forest	0.7956	2.252	1.783	2.25	*n* = 100, d = 7
Gradient boosting	0.8589	1.871	1.479	1.86	*n* = 100, d = 2, lr = 0.1
XGBoost	0.8492	1.934	1.529	1.93	*n* = 100, d = 2, lr = 0.1
SVR	0.7842	2.314	1.752	2.20	rbf, C = 10

The bold values indicate the best performance of each evaluation metric in this table.

Lasso Regression achieves the highest *R*^2^ = 0.9443 among all models tested, explaining approximately 94.4% of the variance in team scoring across 15 test seasons. The four regularized linear models (Lasso, ElasticNet, Ridge, and Linear Regression) cluster tightly at *R*^2^>0.942, substantially outperforming the ensemble tree models. Random Forest (*R*^2^ = 0.796) and SVR (*R*^2^ = 0.784) perform notably worse, likely due to overfitting on the limited training samples. As shown in Table 5, the differences among the regularized linear models are not statistically significant for the closest competitors, indicating that the primary advantage lies in the linear family as a whole rather than in any single specification.

**Figure 3:**
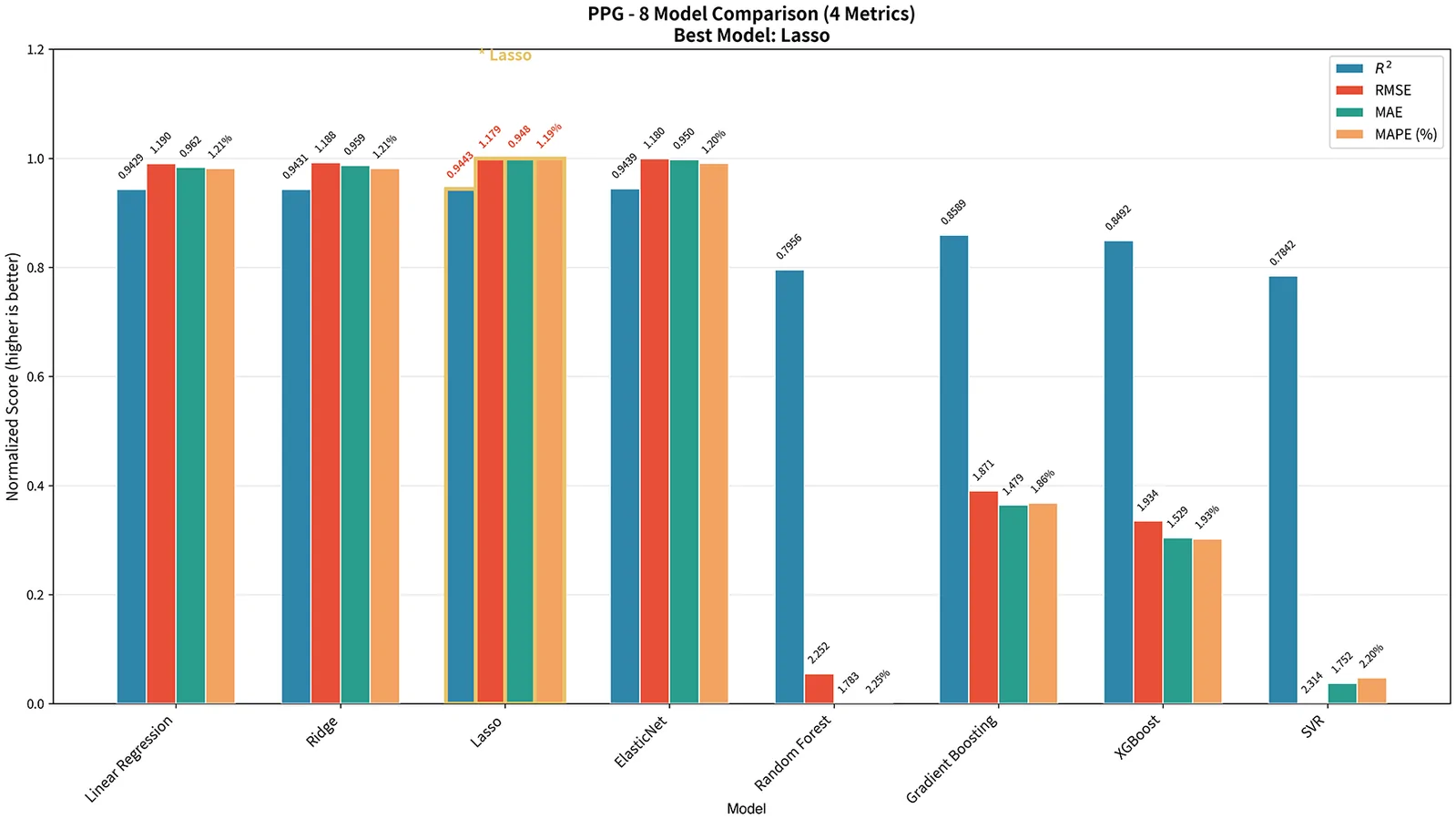
PPG modeling model comparison across eight regression algorithms. Lasso Regression achieves the highest *R*^2^ = 0.9443, RMSE = 1.179, MAE = 0.948, and MAPE = 1.19%. The four regularized linear models (Lasso, ElasticNet, Ridge, and Linear Regression) cluster closely at the top, substantially outperforming ensemble tree methods and SVR.

Figure 3 visualizes the model comparison across all four metrics. As shown in the figure, the four regularized linear models achieve nearly identical and superior performance across R^2^, RMSE, MAE, and MAPE, while the ensemble tree models (Random Forest, Gradient Boosting, and XGBoost) and SVR fall substantially behind. Lasso yields the numerically highest R^2^ (0.9443 vs. 0.9429 for Linear Regression) and the lowest error metrics (RMSE = 1.179, MAE = 0.948). The grouped bar chart also reveals that the performance gap between linear and non-linear models is consistent across all four evaluation dimensions, confirming the robustness of the linear modeling assumption for this task.

#### 3.2.2 Lasso regression coefficients and interpretation

A key advantage of Lasso Regression is its interpretability. The *L*_1_ penalty drives the coefficients of irrelevant features to exactly zero, performing automatic feature selection. Table 7 presents the non-zero coefficients of the PPG Lasso model.

**Table 7:** Lasso coefficients for PPG modeling (sorted by absolute value).

Feature	Coefficient
TS%	+2.594
TRB	+2.031
NRtg	+1.803
Pace	+1.732
Opp_FG%	+1.417
REB_NET	−0.845
AST_TO	+0.828
AST	−0.713
Opp_3P%	+0.355
Q_VOL	−0.154
C_REB_RATE	−0.129
G_3P_REL	−0.083
Q3_SURGE	−0.055
OPP_FG_SUPP	−0.035
POS_BAL	+0.017

Features with zero coefficient (excluded by Lasso): FG%, 3P%, Q4_SHARE.

The Lasso model retains 15 of 18 features and excludes 3 (FG%, 3P%, and Q4_SHARE). The dominance of TS% (True Shooting Percentage) as the strongest predictor is consistent with basketball analytics principles: efficient scoring is the primary driver of points per game. The positive coefficient for Pace is associated with faster-paced teams tending to score more. Several coefficients warrant careful interpretation from a basketball perspective.

The negative coefficient for AST (−0.713) does not imply a causal relationship. Rather, it reflects a conditional association within the specific model specification. In a similar way, the negative coefficient for REB_NET (−0.845) is a hypothesis-generating observation, not a causal claim.

The positive coefficient for Opp_FG% (+1.417) captures a “pace effect” rather than any causal benefit from poor defense. Higher opponent shooting percentages often correlate with faster-paced games where both teams accumulate more possessions.

##### 3.2.2.1 Reconciling Lasso coefficient and ablation result for AST_TO

Table 7 shows that AST_TO receives a positive coefficient (+0.828) in the Lasso model, which is associated with a beneficial relationship with PPG. However, the ablation experiment reveals that removing AST_TO actually improves model performance (Δ*R*^2^ = −0.0011, i.e., *R*^2^ increases from 0.9443 to 0.9454). This apparent contradiction arises because Lasso, while performing feature selection, may still retain features that are marginally useful in combination with others but become redundant when stronger predictors are present. AST_TO is moderately correlated with TS% (*r* = 0.51) and NRtg (*r* = 0.48). Once TS% and NRtg are included, the unique information from AST_TO is minimal; its positive coefficient in the full model is a result of regularization-induced shrinkage that does not fully eliminate it, but ablation, which directly measures marginal utility, correctly identifies its redundancy. This highlights the value of systematic ablation as a complement to automatic feature selection: regularization can retain features that are not truly beneficial, whereas ablation directly quantifies their marginal contribution.

**Figure 4:**
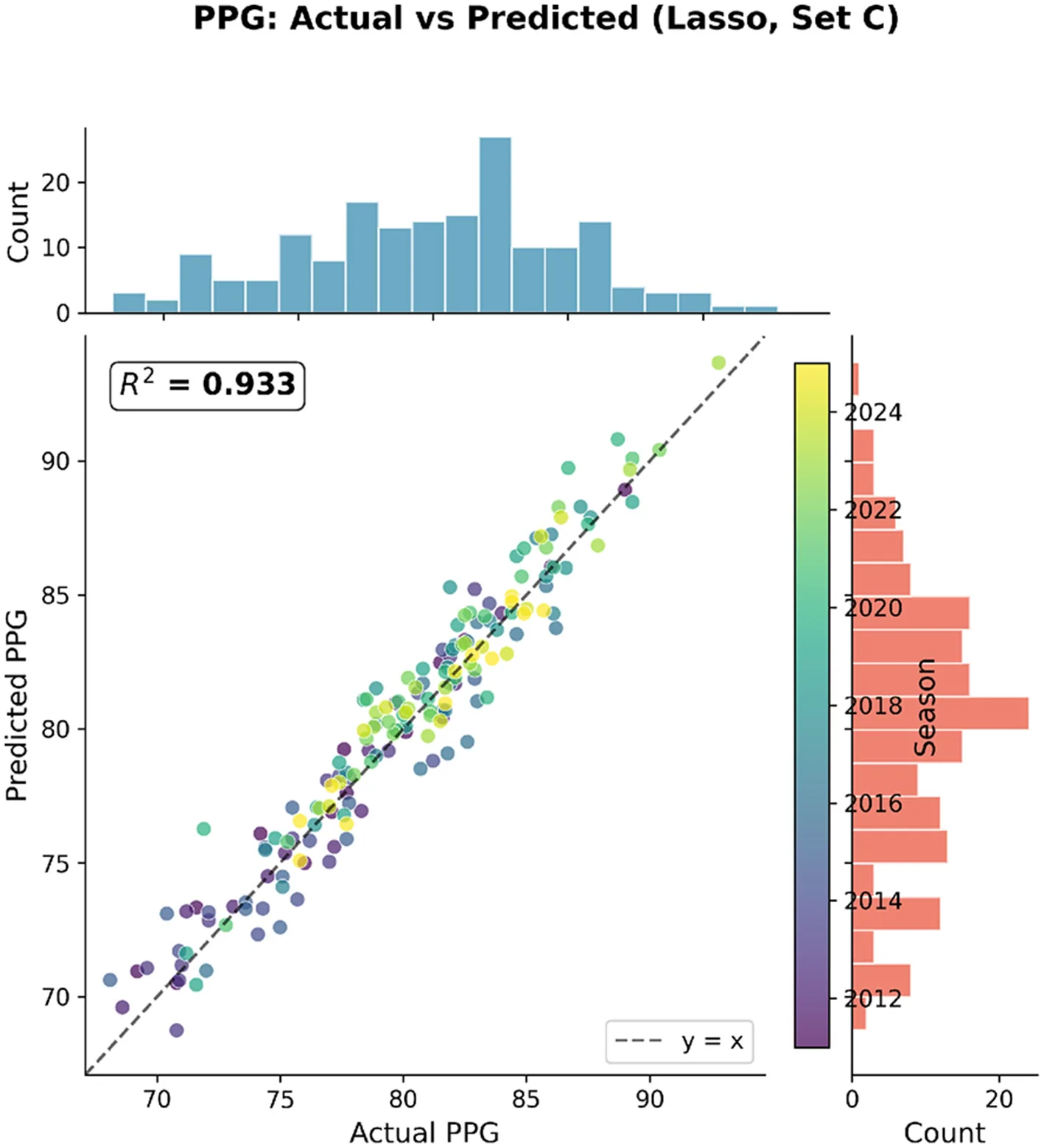
Actual vs. estimated PPG values (Lasso, Set C). Points are colored by test season. The dashed line represents perfect estimation (*y* = ŷ). The model achieves *R*^2^ = 0.9443 across 15 test seasons (2011–2025).

Figure 4 presents the actual vs. estimated PPG values, demonstrating the strong agreement between the Lasso model's estimates and observed team scoring. The scatter points cluster tightly along the *y* = ŷ diagonal, with no systematic deviation across the range of observed PPG values (approximately 70–90 points). The marginal histograms further confirm that the distributions of actual and predicted values are well-matched, and the color gradient by test season shows no temporal drift in estimation accuracy.

The standardized regression equation is:


$$\begin{array}{l} {\hat{y}}_{PPG}=\;2.594\cdot z_{TS\%}+2.031\cdot z_{TRB}+1.803\cdot z_{NRtg}+1.732\cdot z_{Pace}\\ \;+1.417\cdot z_{Opp\_FG\%}+0.828\cdot z_{AST\_TO}+0.355\cdot z_{Opp\_3P\%}\\ \;+0.017\cdot z_{POS\_BAL}-0.845\cdot z_{REB\_NET}-0.713\cdot z_{AST}\\ \;-0.154\cdot z_{Q\_VOL}-0.129\cdot z_{C\_REB\_RATE}-0.083\cdot z_{G\_3P\_REL}\\ \;-0.055\cdot z_{Q3\_SURGE}-0.035\cdot z_{OPP\_FG\_SUPP} \end{array}$$ (1)

**Figure 5:**
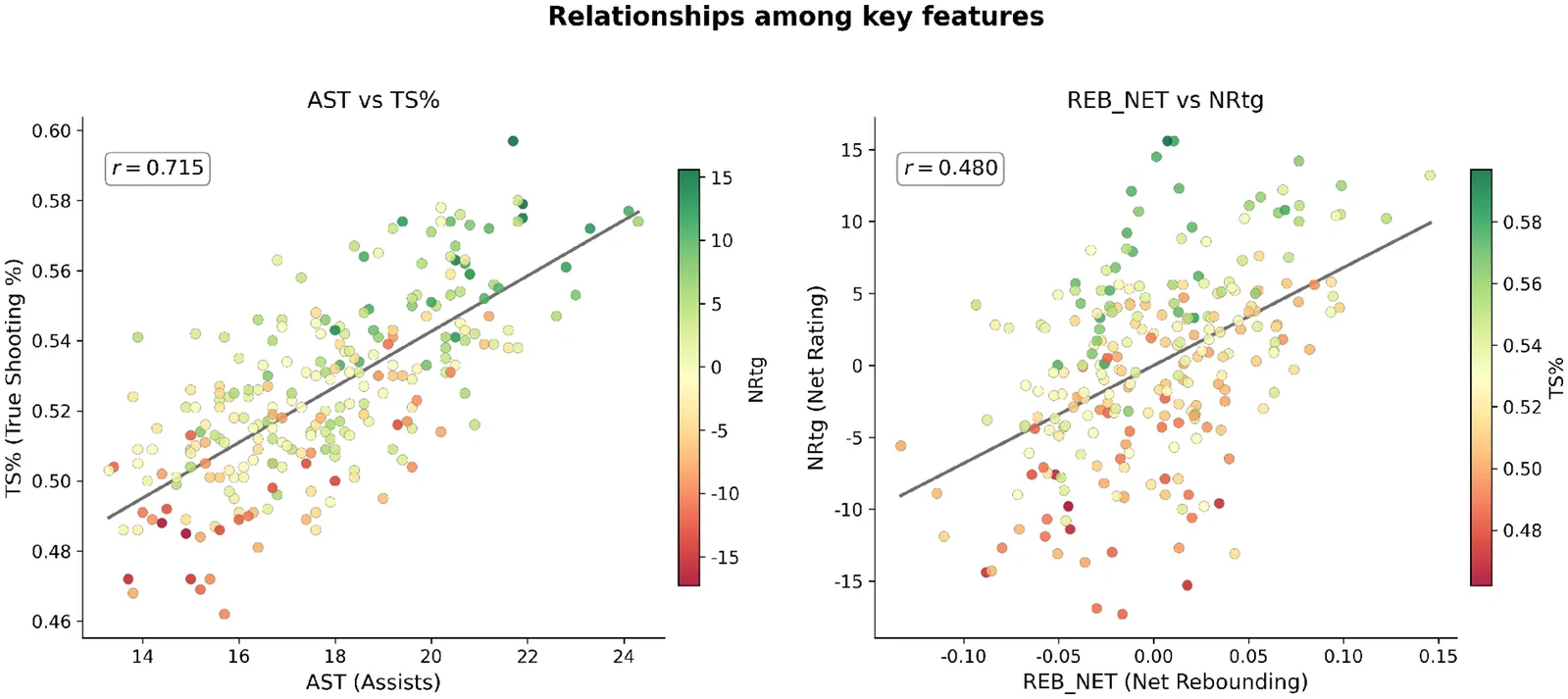
Relationships among key features. **(Left)** AST is strongly positively correlated with TS% (*r* = 0.715), indicating that high-assist teams are generally high-efficiency teams. **(Right)** REB_NET is moderately positively correlated with NRtg (*r* = 0.480), indicating that rebounding advantages generally benefit team performance.

This interpretation is supported by the strong positive correlation between AST and TS% (*r* = 0.715, Figure 5), indicating that high-assist teams are generally high-efficiency teams. Table 8 further demonstrates that AST is positively associated with overall team quality in raw data (NRtg increases monotonically from −3.65 to +4.40 across quartiles), confirming that the negative coefficient reflects variance allocation in regularized regression rather than a true negative relationship. Figure 5 also shows that REB_NET is moderately positively correlated with NRtg (*r* = 0.480), indicating that rebounding advantages generally benefit team performance, but this relationship is overshadowed by TS% in the regression model.

**Table 8:** Quartile analysis of assists (AST) and team performance metrics.

AST Quartile	*n*	Pace	TS%	NRtg	FG%
Q1 (lowest)	62	76.15	0.504	−3.648	0.413
Q2	65	76.96	0.518	−0.826	0.429
Q3	61	77.80	0.529	0.320	0.439
Q4 (highest)	59	78.70	0.553	**4.395**	0.455

The bold values indicate the best performance of each evaluation metric in this table.

**Figure 6:**
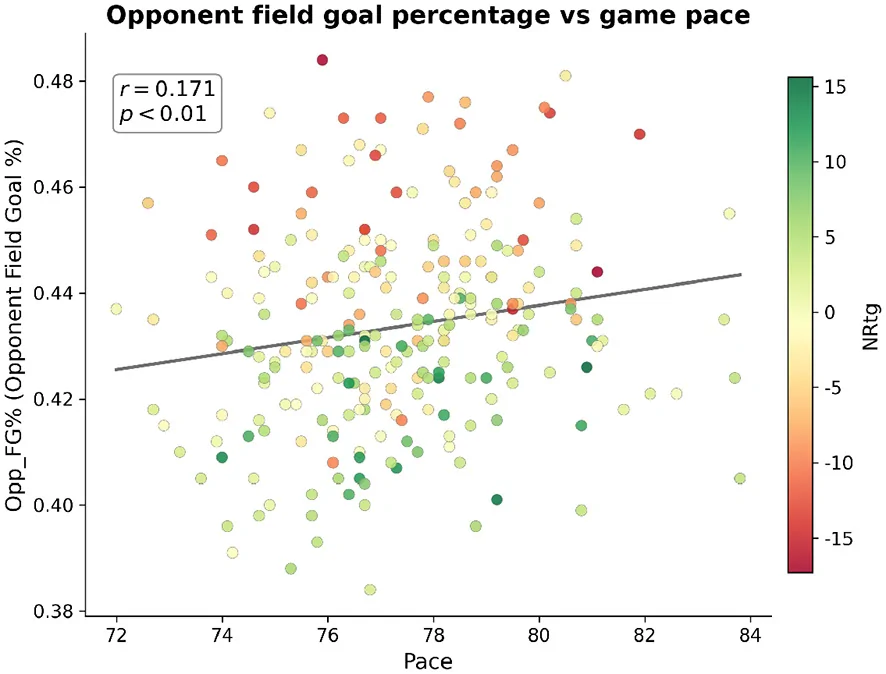
Opponent field goal percentage vs game pace. Opp_FG% is weakly but significantly positively correlated with Pace (*r* = 0.171, *p* < 0.01), supporting the interpretation that higher opponent shooting percentages reflect faster-paced games with increased possessions for both teams. Color indicates NRtg (red = negative, green = positive).

Figure 6 confirms this relationship: Opp_FG% is weakly but significantly positively correlated with Pace (*r* = 0.171, *p* < 0.01). Additionally, when opponents score efficiently, teams may accelerate their offensive tempo in transition, creating additional scoring opportunities. The positive coefficient reflects this residual pace-related variance after NRtg absorbs the defensive efficiency component (Opp_FG% is strongly negatively correlated with NRtg, *r* = −0.571).

#### 3.2.3 Ablation experiment for PPG

To validate the contribution of each engineered feature, we conduct a leave-one-out ablation experiment: each of the nine engineered indicators is removed one at a time from Set C, and the Lasso model is retrained using the same expanding-window folds. The change in *R*^2^ ($\Delta R^{2}=R_{\text{full}}^{2}-R_{\text{without}}^{2}$) measures each feature's marginal contribution, where positive values indicate that removing the feature harms performance (i.e., the feature is helpful). Table 9 presents the complete ablation results.

**Table 9:** PPG ablation results for all nine engineered features.

Feature	*R*^2^ (without)	*ΔR* ^2^	Contribution
Positive contributions (feature helps):			
REB_NET	0.9367	+0.0076	Positive
Q_VOL	0.9430	+0.0013	Positive
C_REB_RATE	0.9438	+0.0005	Positive
Negative contributions (feature harms):			
POS_BAL	0.9447	−0.0004	Negative
Q3_SURGE	0.9449	−0.0006	Negative
Q4_SHARE	0.9451	−0.0008	Negative
AST_TO	0.9454	−0.0011	Negative
G_3P_REL	0.9454	−0.0011	Negative
OPP_FG_SUPP	0.9456	−0.0013	Negative

Full model (Set C, 18 features): *R*^2^ = 0.9443.

**Figure 7:**
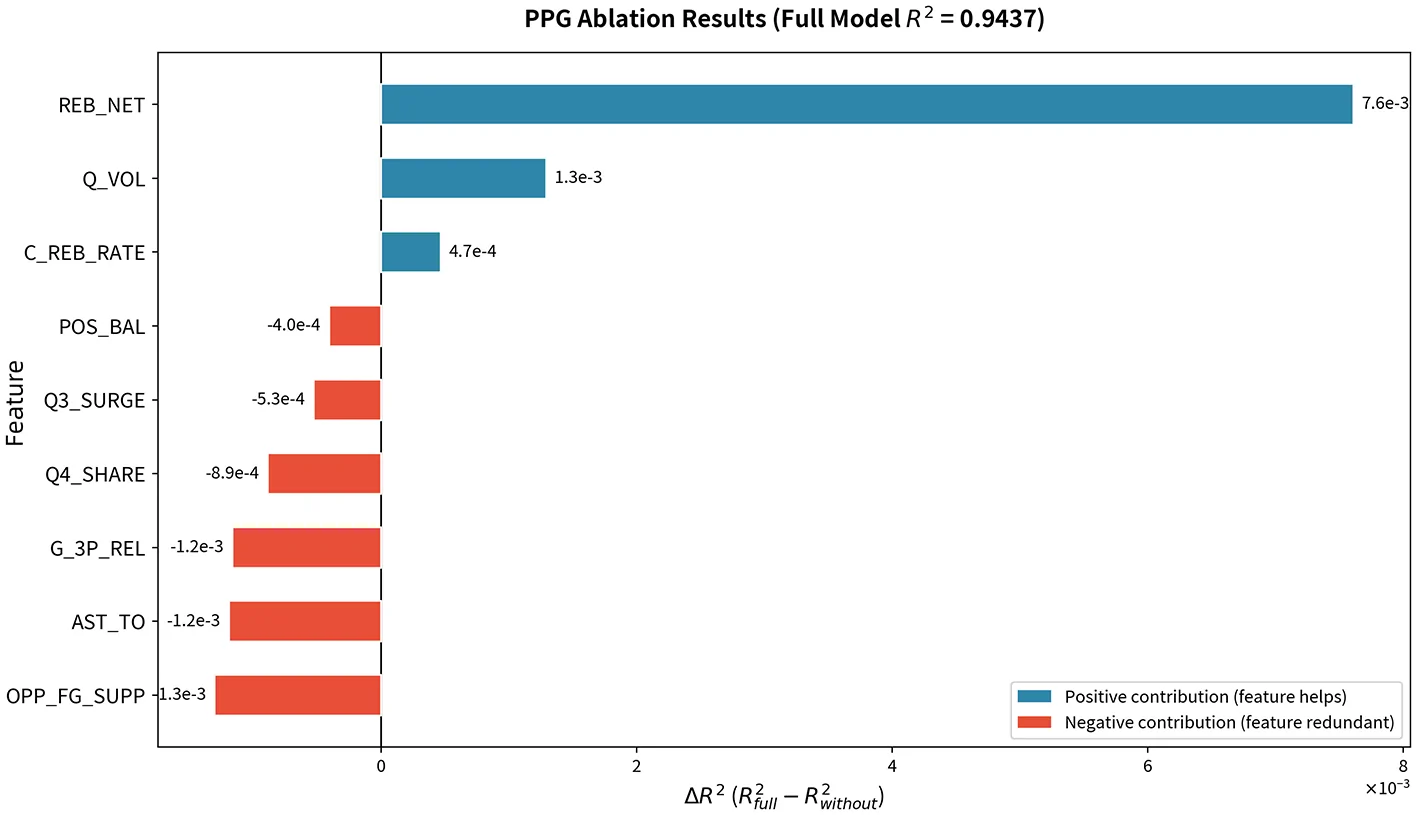
PPG modeling: ablation results for all nine engineered features. Three features (REB_NET, Q_VOL, and C_REB_RATE) show positive contributions; six features show negative contributions, indicating redundancy with existing predictors.

Figure 7 visualizes the ablation results. The horizontal bar chart clearly separates the nine engineered features into two groups: three with positive Δ*R*^2^ (blue bars, indicating helpful features) and six with negative Δ*R*^2^ (red bars, indicating redundant features). REB_NET dominates with Δ*R*^2^ = +0.0076, an order of magnitude larger than the next best feature (Q_VOL, +0.0013). The six negative-contribution features show similar magnitudes of redundancy (Δ*R*^2^≈−0.0004 to −0.0013), suggesting that they capture information already well-represented by the base features. Only three of the nine engineered features show positive Δ*R*^2^, indicating that they provide unique predictive information beyond the base features. REB_NET (Rebounding Net) contributes the most (Δ*R*^2^ = +0.0076), suggesting that rebounding dominance captures scoring potential not fully reflected in standard efficiency metrics. Q_VOL (Quarterly Scoring Volatility) and C_REB_RATE (Center Rebound Contribution Rate) provide smaller but detectable contributions (Δ*R*^2^ = +0.0013 and +0.0005, respectively).

Notably, six engineered features show negative contributions, meaning that removing them *improves* model performance. This indicates redundancy with existing predictors rather than lack of predictive signal: these features capture information already represented by the base features (FG%, TS%, NRtg, etc.). This finding has important implications for feature engineering: **more features do not always yield better estimation**.

**Figure 8:**
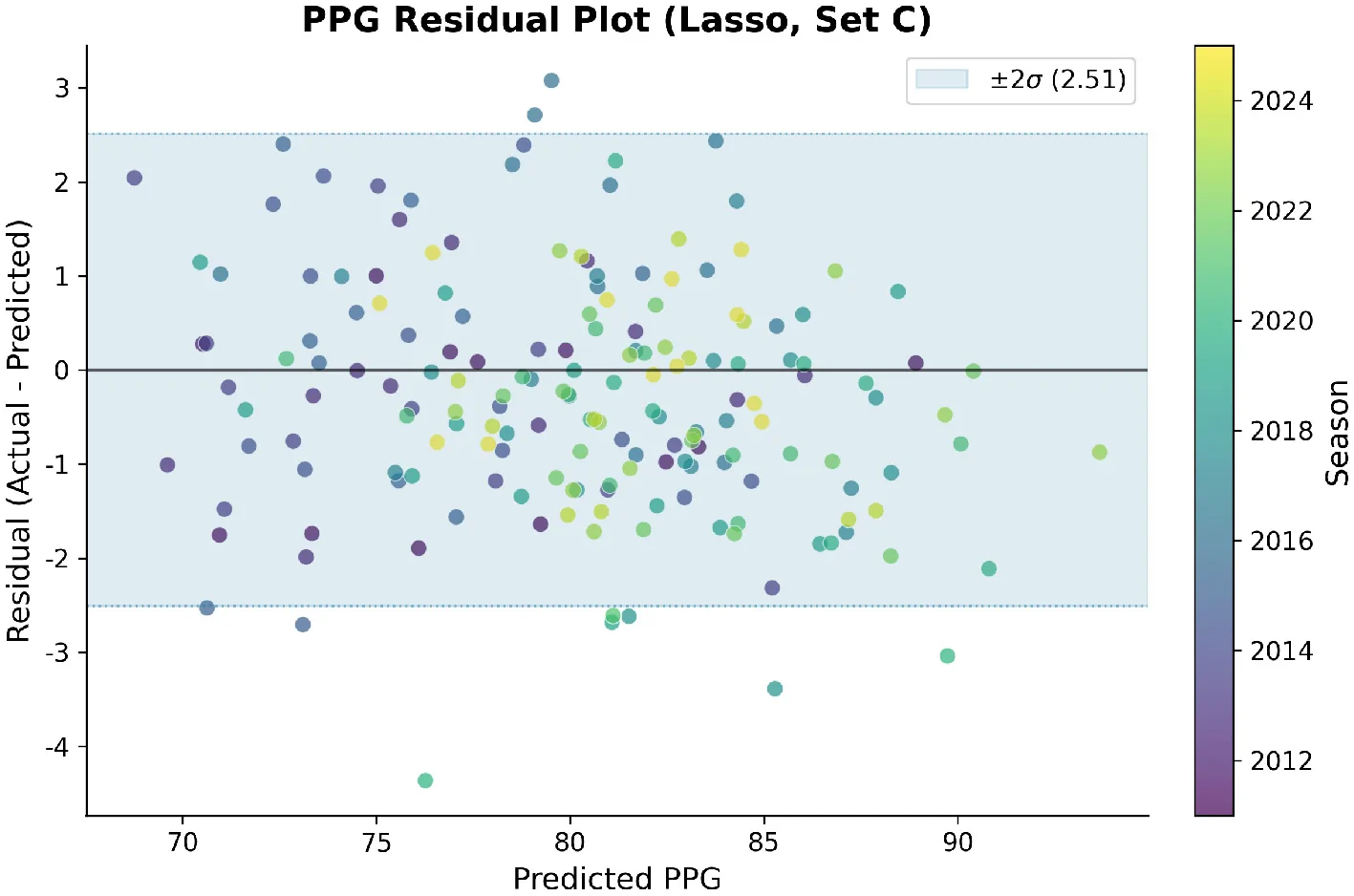
PPG residual plot (Lasso, Set C). Residuals are randomly distributed around zero with no systematic patterns, confirming the adequacy of the linear model assumption. The shaded band represents the ±2σ confidence interval.

Figure 8 shows the residual distribution, confirming that estimation errors are randomly distributed around zero with no systematic patterns, validating the linear model assumption. The ±2σ confidence band contains nearly all observations, and no heteroscedasticity is visible across the predicted range.

**Figure 9:**
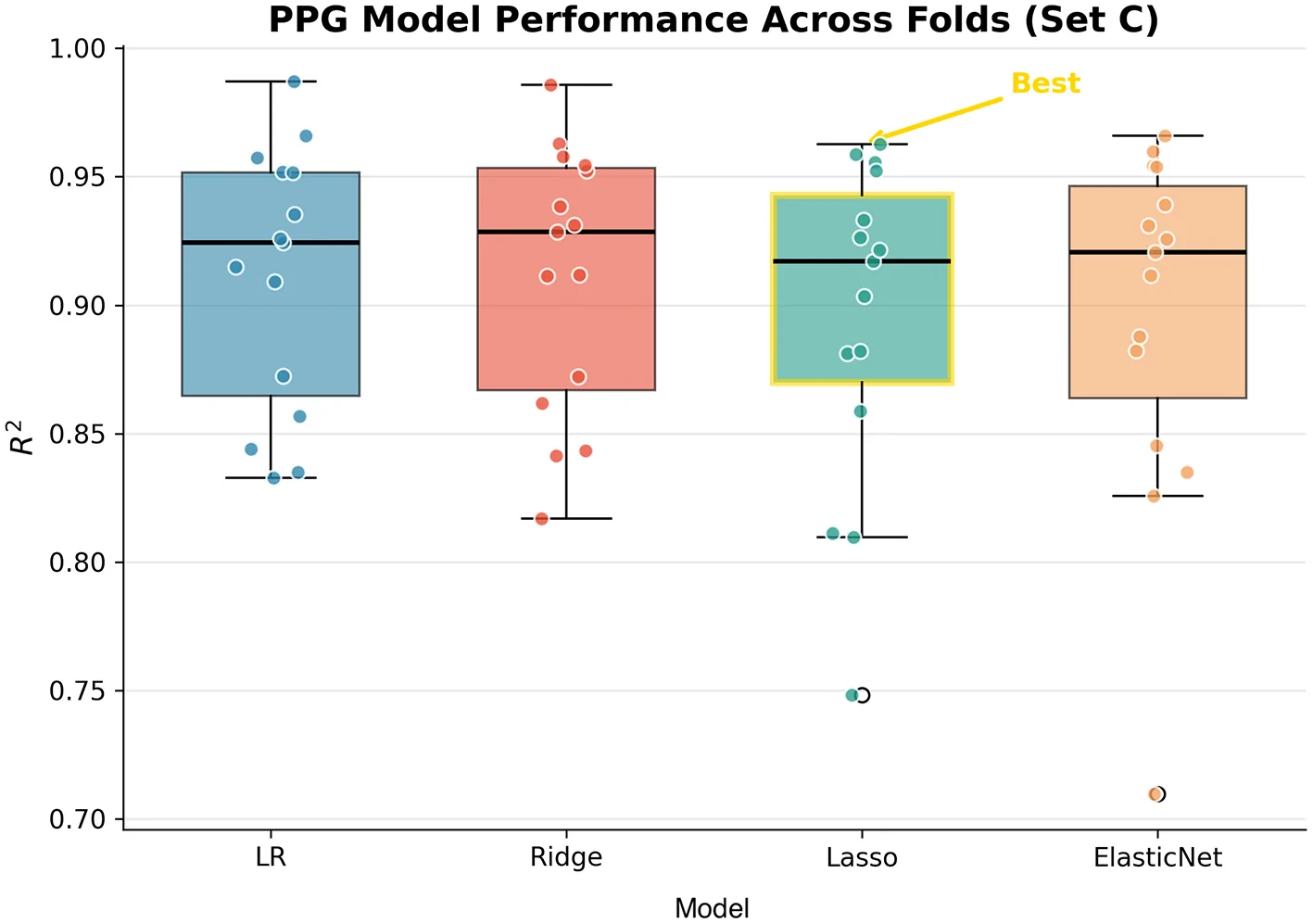
Cross-fold *R*^2^ distribution for PPG modeling across eight models. Each box represents the distribution of fold-wise *R*^2^ values across 15 test seasons (2011–2025). Lasso (highlighted) achieves the highest median *R*^2^ = 0.9443 with relatively low variability.

Figure 9 presents the cross-fold variability in model performance. The boxplot reveals that Lasso not only achieves the highest median fold-wise *R*^2^ but also maintains relatively low interquartile variability, indicating stable performance across different test seasons rather than reliance on any single favorable fold.

#### 3.2.4 Coefficient stability across folds

To assess the robustness of the Lasso coefficient estimates, we examined the sign consistency of each feature's coefficient across the 15 expanding-window folds. The core features, TS%, TRB, Pace, NRtg, Opp_FG%, AST_TO, and REB_NET, exhibited 100% sign consistency across all folds, indicating highly stable directional relationships. The AST coefficient was negative in all 15 folds (100%), confirming a robust conditional association. Among the engineered features, Q3_SURGE and Q4_SHARE showed lower sign consistency (66.7% and 40%, respectively), indicating that their coefficient directions are sensitive to the training composition. These results suggest that while the core feature–target relationships are robust, coefficient estimates for certain engineered features should be interpreted with caution, and their primary value lies in marginal predictive contribution rather than in directional interpretation.

#### 3.2.5 Reduced-specification experiment

To evaluate the extent to which the high *R*^2^ values depend on definitional variables, features that share mathematical definitions with the target, we conducted reduced-specification experiments. For WIN%, removing NRtg (which is partially definitional, as win percentage is closely linked to net efficiency) and using only Set A yields *R*^2^ = 0.691, a substantial drop of 0.238 from the Set C value of 0.9287. For PPG, removing Pace and TS% (which directly influence points accumulation) yields *R*^2^ = 0.790, a drop of 0.154 from the Set C value of 0.9443. These results confirm that a portion of the high *R*^2^ is attributable to definitional overlap between features and targets. However, the reduced models still explain a substantial proportion of variance (69%–79%), indicating that the overall framework retains meaningful explanatory power even without the most definitionally related predictors.

### 3.3 WIN% modeling results

#### 3.3.1 Model comparison

Table 10 presents the WIN% modeling performance across eight models.

**Table 10:** WIN% modeling performance across eight models.

Model	*R* ^2^	RMSE	MAE	MAPE	Best params
Linear regression	0.9197	0.050	0.042	9.86	–
Ridge	0.9207	0.050	0.041	9.74	α = 1.0
Lasso	0.9286	0.047	0.039	9.29	α = 0.01
**ElasticNet**	**0.9287**	**0.047**	**0.039**	9.33	α = 0.01, *l*_1_ = 0.5
Random forest	0.9179	0.051	0.042	11.08	*n* = 200, d = 3
Gradient boosting	0.9165	0.051	0.041	10.95	*n* = 50, d = 2, lr = 0.1
XGBoost	0.9153	0.052	0.041	10.73	*n* = 50, d = 2, lr = 0.1
SVR	0.6118	0.110	0.086	24.00	rbf, C = 1

The bold values indicate the best performance of each evaluation metric in this table.

ElasticNet Regression achieves the best *R*^2^ = 0.9287 and RMSE = 0.047. Notably, Lasso achieves a marginally better MAE (differing by 0.0002 at higher precision, though both round to 0.039) and MAPE (9.29% vs. 9.33%). This minor discrepancy arises because *R*^2^ is optimized on squared errors while MAE/MAPE are optimized on absolute errors; ElasticNet's *L*_1_+*L*_2_ combination slightly favors squared-error optimization. Since the differences are negligible (MAPE differs by 0.04 percentage points), we select ElasticNet as the optimal model based on the primary criterion of *R*^2^. SVR performs catastrophically (*R*^2^ = 0.612), likely due to the sensitivity of the RBF kernel to feature scaling and the limited sample size. The ensemble tree models perform respectably (*R*^2^≈0.915–0.918) but are consistently outperformed by the regularized linear models.

**Figure 10:**
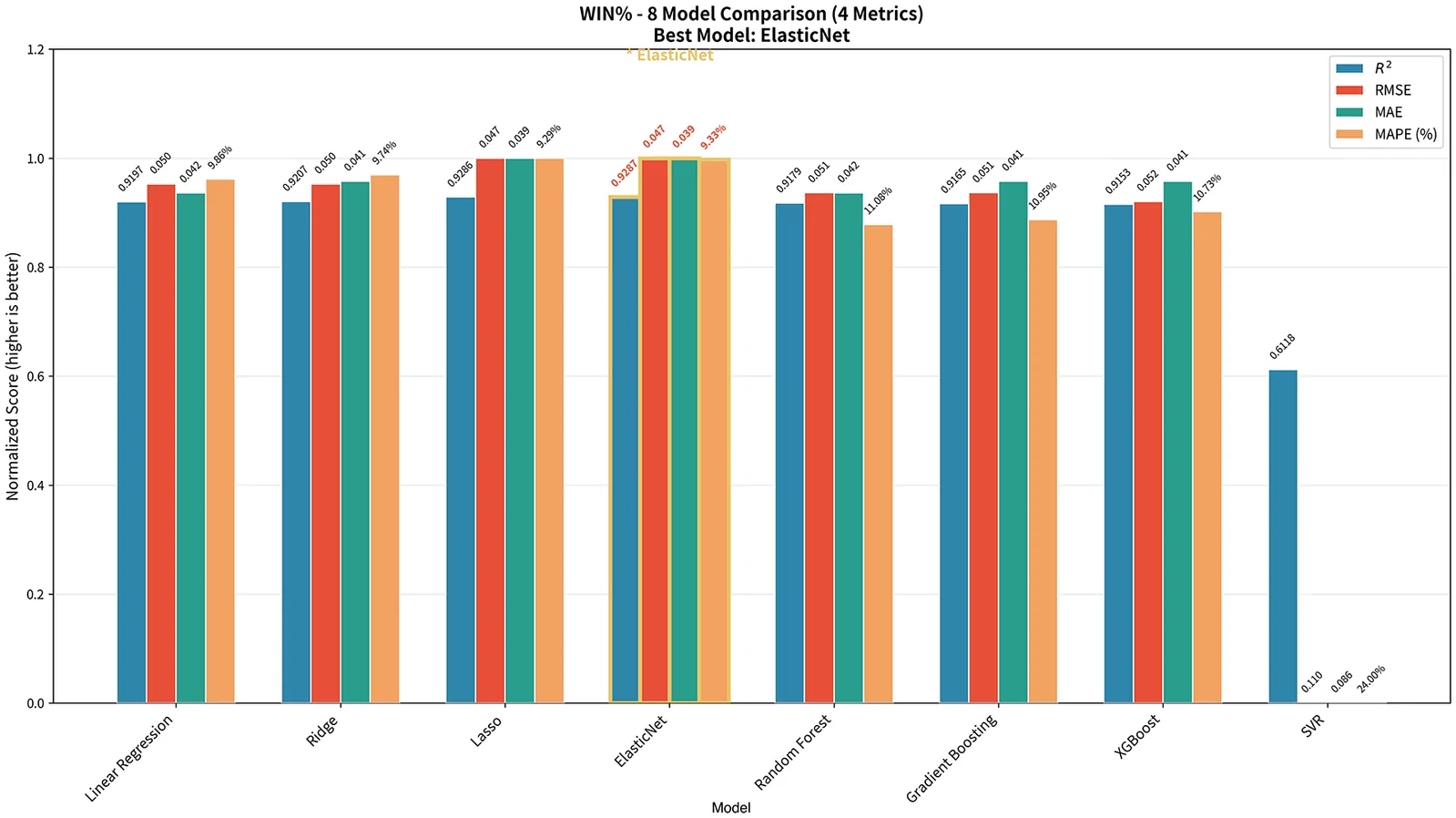
WIN% modeling performance comparison across four evaluation metrics. ElasticNet Regression achieves the highest *R*^2^ = 0.9287, RMSE = 0.047, and MAE = 0.039. The four regularized linear models substantially outperform ensemble tree methods and SVR.

Figure 10 visualizes the model comparison. The grouped bar chart shows a pattern similar to PPG modeling: the four regularized linear models cluster at the top across all four metrics, while SVR (*R*^2^ = 0.612) performs catastrophically. A notable difference from PPG is that the ensemble tree models achieve competitive *R*^2^ values (≈0.915–0.918) but are outperformed on error metrics (MAE, MAPE) by the linear models, indicating that while tree models capture the general trend, they produce larger individual estimation errors.

#### 3.3.2 ElasticNet coefficients and interpretation

The ElasticNet model for WIN% modeling exhibits strong sparsity, retaining only 6 of 18 features (Table 11).

**Table 11:** Non-zero ElasticNet coefficients for WIN% modeling.

Feature	Coefficient
NRtg	+0.146
OPP_FG_SUPP	+0.011
TS%	+0.002
3P%	+0.002
Opp_FG%	−0.001
Opp_3P%	−0.001

Features with zero coefficient (excluded).

FG%, TRB, Pace, AST, Q3_SURGE, Q4_SHARE, C_REB_RATE, Q_VOL, POS_BAL, G_3P_REL, REB_NET, AST_TO.

The overwhelming dominance of NRtg (coefficient = 0.146, more than 13 × the next largest) confirms that net rating, the difference between offensive and defensive efficiency, is the single most important determinant of win percentage. This finding aligns with established basketball analytics: teams that outscore opponents per possession win games, regardless of pace or style.

**Figure 11:**
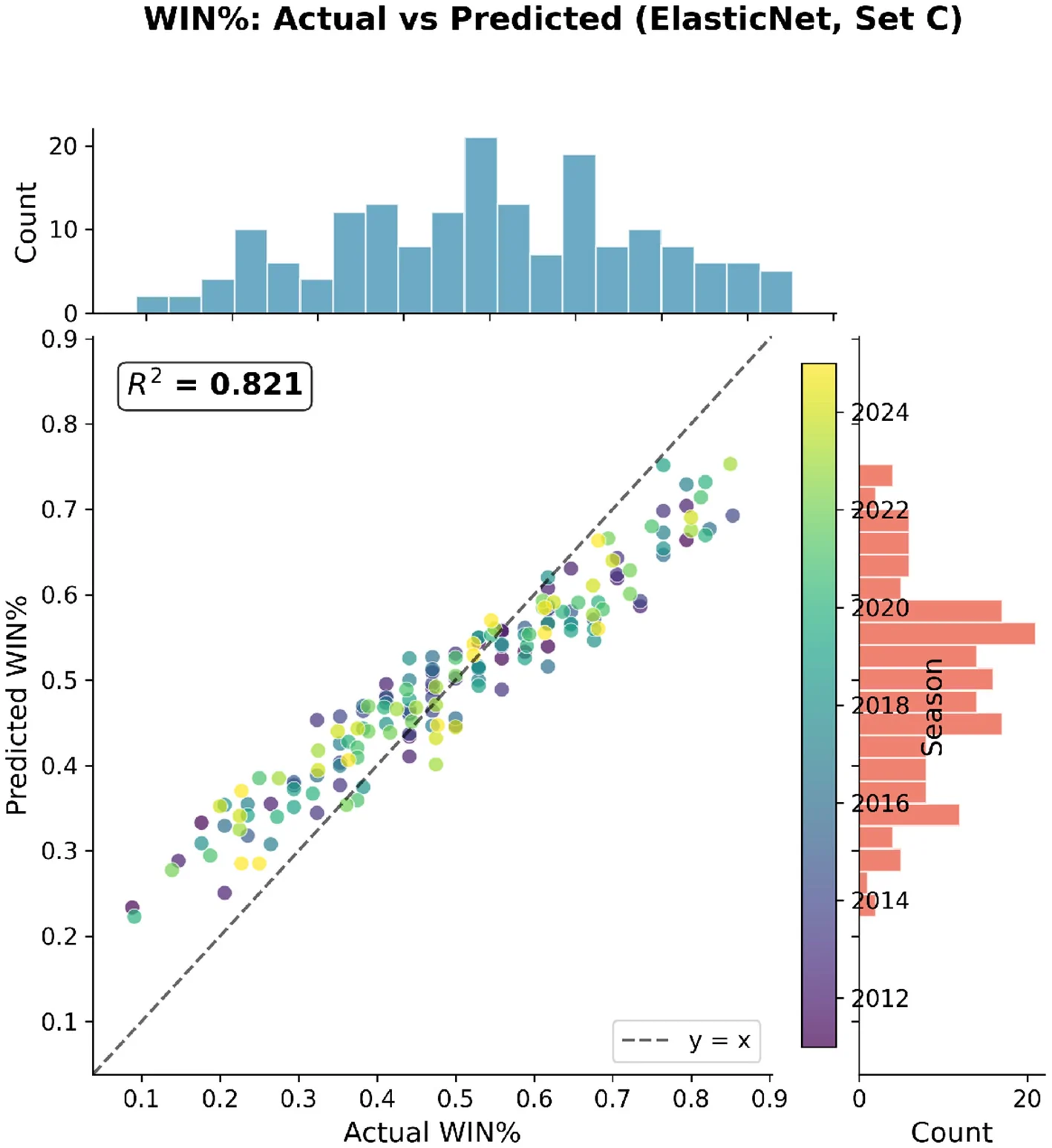
Actual vs. estimated WIN% values (ElasticNet, Set C). The model achieves *R*^2^ = 0.9287 across 15 test seasons.

Figure 11 presents the actual vs. estimated WIN% values. The scatter plot shows strong agreement between estimates and observations, with points tightly distributed along the diagonal. Compared to the PPG scatter plot, the WIN% estimates exhibit slightly wider dispersion for extreme values (very high or very low win percentages), which is expected given that win percentage is bounded at [0, 1] and influenced by factors beyond box-score statistics.

**Figure 12:**
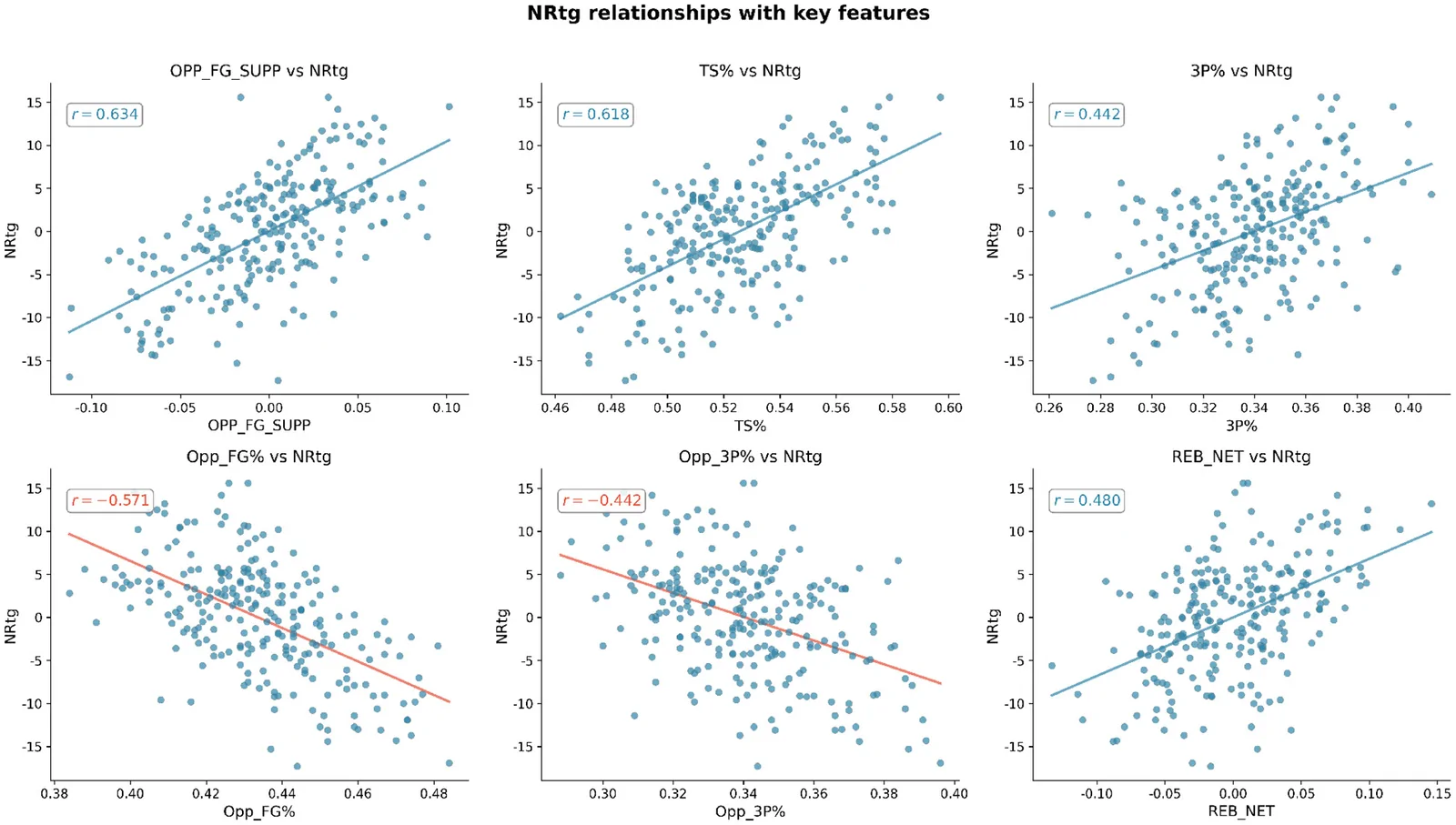
Relationships between NRtg and key features. Top row: OPP_FG_SUPP (*r* = 0.634), TS% (*r* = 0.618), and 3P% (*r* = 0.442) are positively correlated with NRtg. Bottom row: Opp_FG% (*r* = −0.571) and Opp_3P% (*r* = −0.442) are negatively correlated with NRtg. REB_NET shows moderate positive correlation (*r* = 0.480).

The retention of OPP_FG_SUPP (+0.011) warrants particular attention. Although NRtg already captures overall efficiency, OPP_FG_SUPP provides specific information about defensive pressure that NRtg does not fully encompass. Correlation analysis (Figure 12) reveals that OPP_FG_SUPP is strongly positively correlated with NRtg (*r* = 0.634), while opponent shooting percentages (Opp_FG%, Opp_3P%) are strongly negatively correlated with NRtg (*r* = −0.571 and −0.442, respectively). This pattern indicates that OPP_FG_SUPP captures a distinct defensive dimension, the ability to suppress opponent shooting relative to league average, that contributes marginally but meaningfully to win estimation beyond raw net efficiency.

The standardized regression equation is:


$$\begin{array}{l} {\hat{y}}_{WIN\%}=0.146\cdot z_{NRtg}+0.011\cdot z_{OPP\_FG\_SUPP}+0.002\cdot z_{TS\%}\\ \;+0.002\cdot z_{3P\%}-0.001\cdot z_{Opp\_FG\%}-0.001\cdot z_{Opp\_3P\%} \end{array}$$ (2)

The exclusion of 12 features by ElasticNet reflects the near-univariate nature of win percentage modeling. Features such as AST, TRB, Pace, and REB_NET, which showed predictive value for PPG, are absorbed by NRtg in the WIN% model. This is consistent with the theoretical understanding that win percentage is fundamentally determined by per-possession scoring differential rather than stylistic factors like pace or specific statistical accumulations.

#### 3.3.3 Ablation experiment for WIN%

Following the same leave-one-out procedure, we evaluate each engineered feature's contribution to WIN% modeling using ElasticNet. Table 12 presents the complete results.

**Table 12:** WIN% ablation results for all nine engineered features.

Feature	*R*^2^ (without)	*ΔR* ^2^	Contribution
Positive contributions (feature helps):			
OPP_FG_SUPP	0.9284	+0.0003	Positive
C_REB_RATE	0.9285	+0.0002	Positive
POS_BAL	0.9287	+0.0000	Positive
Q4_SHARE	0.9287	+0.0000	Positive
Negative contributions (feature harms):			
AST_TO	0.9287	−0.0000	Negative
Q3_SURGE	0.9287	−0.0000	Negative
Q_VOL	0.9287	−0.0000	Negative
G_3P_REL	0.9288	−0.0001	Negative
REB_NET	0.9288	−0.0001	Negative

Full model (Set C, 18 features): *R*^2^ = 0.9287.

**Figure 13:**
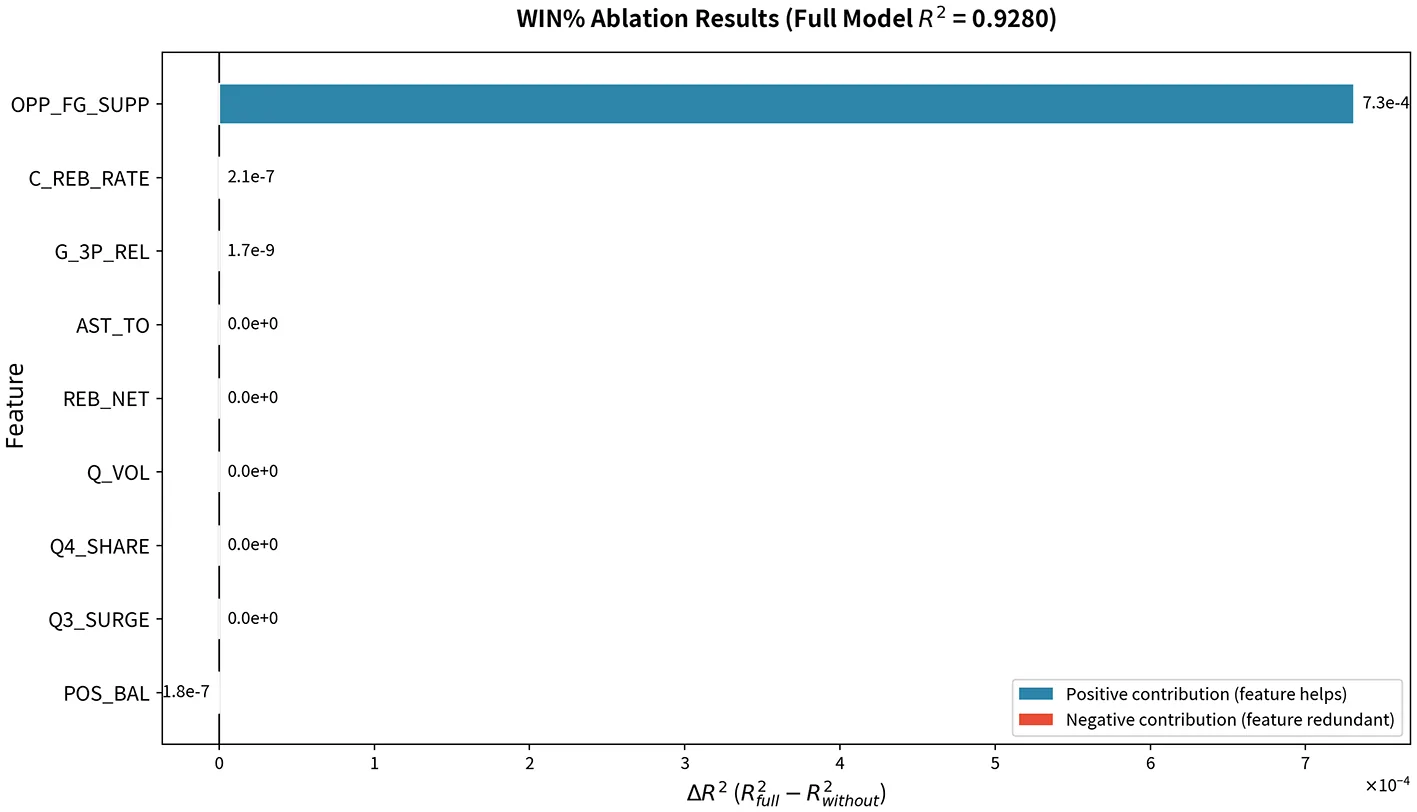
WIN% modeling: ablation results for all nine engineered features. Four features show small positive contributions; five features show negative contributions.

Figure 13 visualizes the WIN% ablation results. In stark contrast to the PPG ablation, all Δ*R*^2^ values are negligibly small (absolute values <0.001), visually confirming that no single engineered feature substantially affects WIN% estimation. The near-zero magnitudes across all nine features indicate that NRtg absorbs virtually all predictive information relevant to win percentage, leaving no room for engineered features to provide incremental value. Four engineered features show positive contributions to WIN% modeling, though magnitudes are very small (Δ*R*^2^ ≤ 0.0003). This is expected given that NRtg already captures the vast majority of win-percentage variance (coefficient 13 × larger than any other feature). The positive contribution of OPP_FG_SUPP aligns with its retention in the ElasticNet model, confirming that defensive pressure provides marginal predictive value beyond net rating.

**Figure 14:**
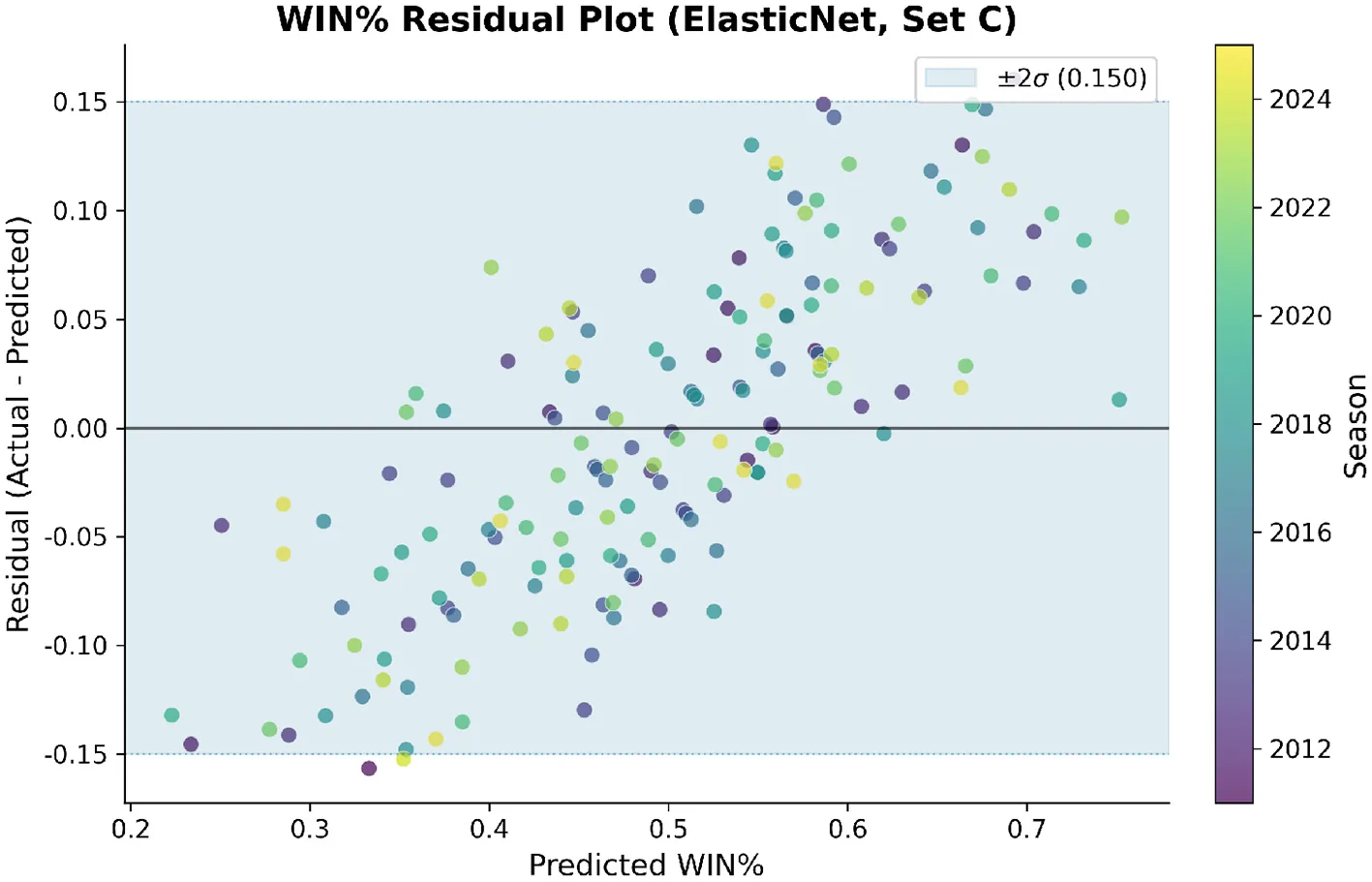
WIN% residual plot (ElasticNet, Set C). The residual distribution confirms the model's adequacy.

Figure 14 presents the residual distribution for the WIN% model. The residual plot shows a pattern similar to PPG: errors are randomly scattered around zero within the ±2σ band, with no visible heteroscedasticity or nonlinearity.

**Figure 15:**
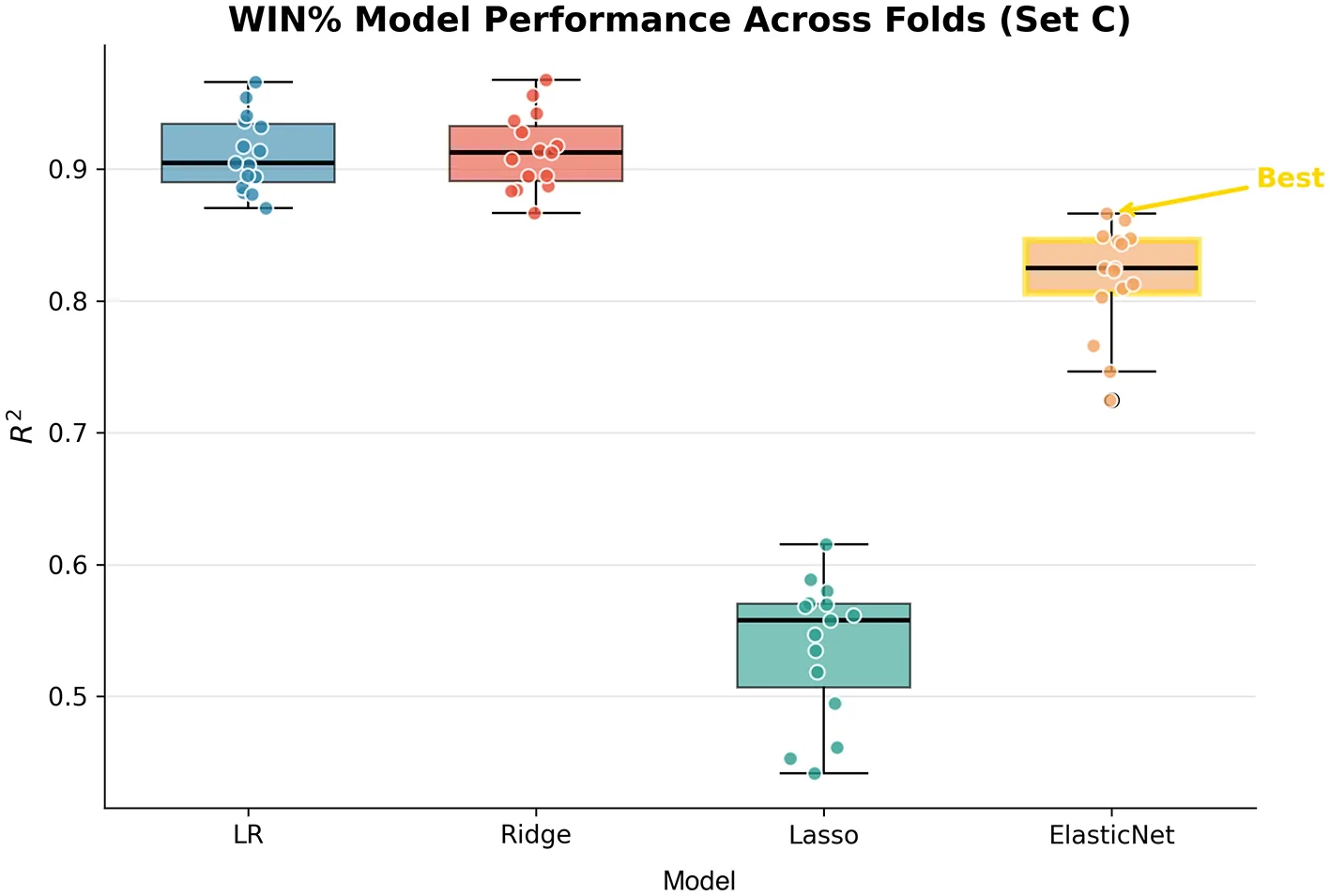
Cross-fold *R*^2^ distribution for WIN% modeling. ElasticNet (highlighted) achieves the highest median *R*^2^ = 0.9287.

Figure 15 demonstrates that ElasticNet achieves the highest median fold-wise *R*^2^ with tight interquartile range, confirming that the model's strong overall performance is not driven by outlier folds.

### 3.4 Team-aware sensitivity analysis

To evaluate whether the models generalize across individual teams rather than being driven by between-team variance, we conducted leave-one-team-out sensitivity analysis. For PPG, the average per-team *R*^2^ was 0.792 (excluding 2 teams with insufficient data), with the majority of teams (14/18 with valid data) achieving *R*^2^>0.85. A few teams with small sample sizes, particularly Houston Comets (*R*^2^ = −0.047, *n* = 3) and Sacramento Monarchs (*R*^2^ = −0.045, *n* = 4), showed negative *R*^2^ values, reflecting insufficient data for reliable within-team estimation rather than model failure. For WIN%, the average per-team *R*^2^ was 0.857 (excluding 2 teams with insufficient data), and all teams with at least 10 observations achieved *R*^2^>0.72. These results suggest that the models are stable for most teams and that their performance is not solely attributable to cross-team variance, though the small-sample caution applies to teams with limited historical data.

## 4 Discussion

**Figure 16:**
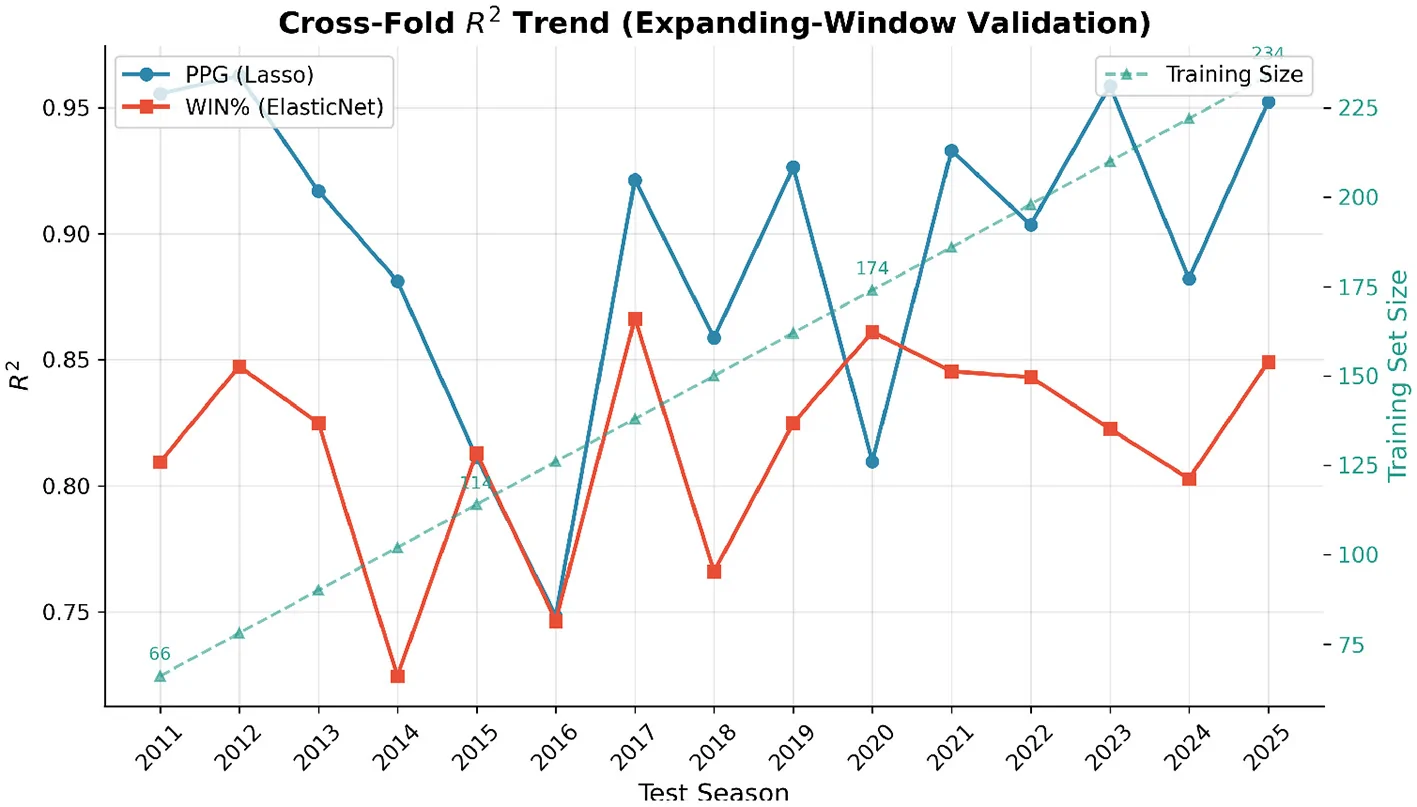
Fold-wise *R*^2^ trend across 15 test seasons (2011–2025). The right axis shows the expanding training set size. Both PPG (Lasso, *R*^2^ = 0.9443) and WIN% (ElasticNet, *R*^2^ = 0.9287) show stable or improving performance as more historical data becomes available, validating the expanding-window approach.

Figure 16 illustrates how estimation accuracy evolves as the training window expands. The left axis plots fold-wise *R*^2^ for both PPG and WIN%, while the right axis shows the growing training set size (from 60 observations in Fold 1 to 228 in Fold 15). Both targets show a general upward trend in *R*^2^ as more historical data becomes available, with PPG exhibiting more pronounced fold-to-fold variability than WIN%. This pattern validates the expanding-window approach: larger training sets provide more representative estimates of feature–target relationships, leading to better generalization.

This study yields four principal findings with implications for basketball analytics methodology.

First, regularized linear models substantially outperform complex alternatives (ensemble trees and SVR) under small-sample constraints, with all pairwise comparisons showing statistically significant advantages over nonlinear models (*p* < 0.001 for all contrasts except Gradient Boosting for WIN%, which was *p* = 0.040). Lasso achieves the numerically highest *R*^2^ = 0.9443 for PPG, and ElasticNet achieves *R*^2^ = 0.9287 for WIN%; however, as shown in Table 5, pairwise comparisons between the top regularized linear models reveal no statistically significant differences for the closest competitors (PPG: Lasso vs. ElasticNet, *p* = 0.619; WIN%: ElasticNet vs. Lasso, *p* = 0.746), indicating that within the linear family, model choice is practically inconsequential for the closest pairs. This contrasts with studies favoring ensemble methods, but aligns with statistical theory: small samples (242 team-seasons) favor simpler models with stronger regularization, and feature-target relationships are predominantly linear.

Second, optimal model structure depends on the target. PPG modeling benefits from Lasso's feature selection (15 retained features), reflecting the multifaceted nature of scoring. WIN% modeling requires only ElasticNet's parsimonious representation (6 retained features), as net efficiency overwhelmingly determines outcomes.

Third, and most notably, **pruning redundant features can be as critical as engineering new ones**. The ablation experiments reveal that only 3 of 9 engineered features contribute positively to PPG modeling; 6 features are redundant and actually harm performance. Removing these redundant features yields Set C′ (12 features), which outperforms the full Set C (18 features) with *R*^2^ = 0.9497 vs. 0.9443.

**Figure 17:**
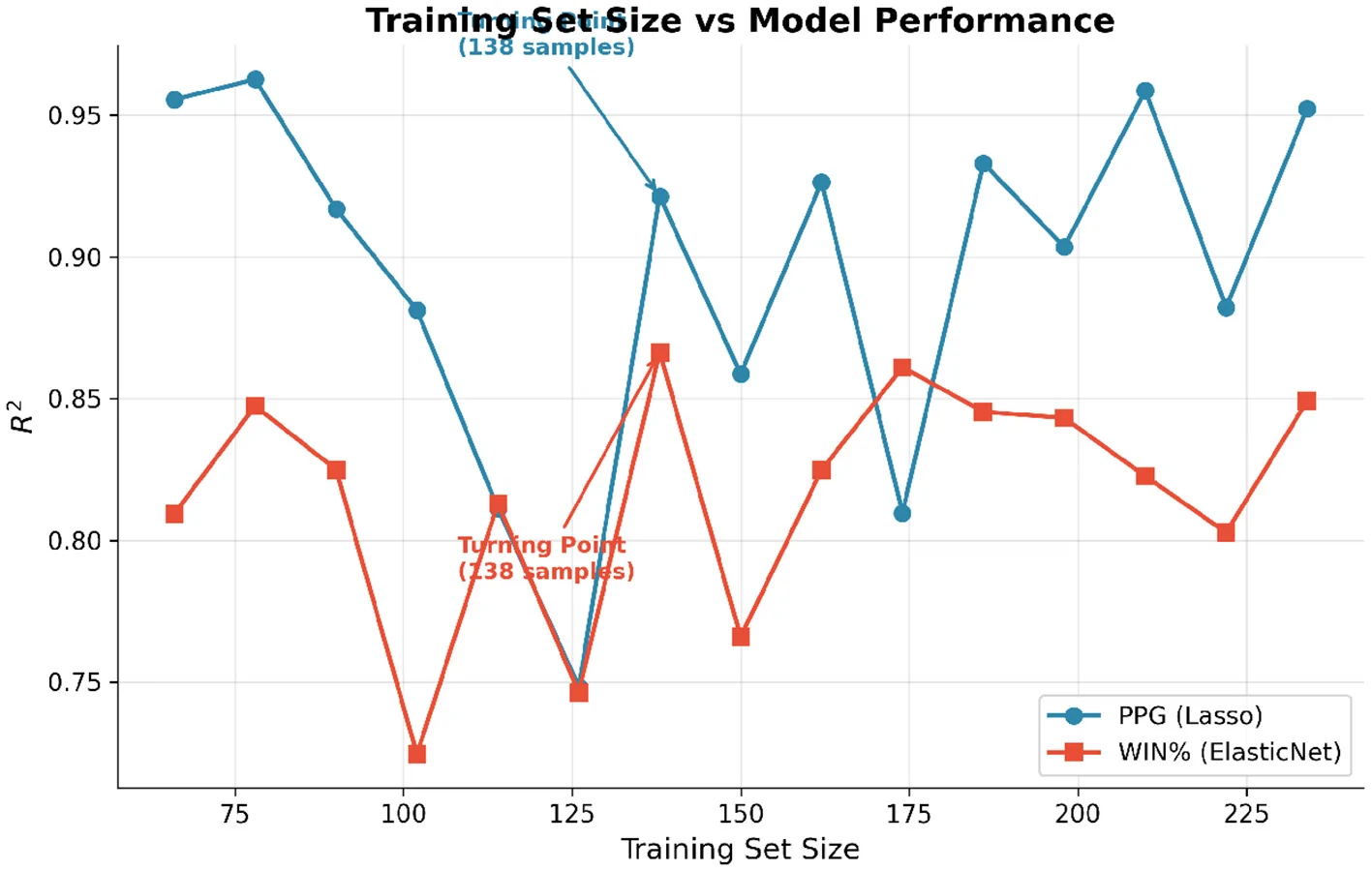
Training set size vs. model performance. Both PPG (Lasso, *R*^2^ = 0.9443) and WIN% (ElasticNet, *R*^2^ = 0.9287) estimation accuracy generally improve as more historical seasons are incorporated into the training window, validating the expanding-window approach.

Figure 17 further demonstrates the relationship between training data availability and model performance. The scatter plot with trend lines confirms a positive association between training set size and *R*^2^ for both targets, with the relationship appearing approximately linear. Notably, even the smallest training sets (60 observations, 5 seasons) achieve *R*^2^>0.90 for both PPG and WIN%, suggesting that the fundamental feature–target relationships are stable and detectable even with limited historical data.

This “less is more” phenomenon has important methodological implications: *adding features without validation can degrade model performance*. We recommend that future feature engineering studies incorporate systematic ablation analysis to identify truly incremental features.

Fourth, and critically for this study, **the value of engineered features is target-dependent**. For PPG, three engineered features (REB_NET, Q_VOL, and C_REB_RATE) provide small but detectable incremental predictive value, but the practical significance of these marginal improvements is limited, and the optimized 12-feature subset (Set C′) achieves the best performance (*R*^2^ = 0.9497). For WIN%, however, the advanced metrics in Set B (particularly NRtg) already capture 92.9% of variance, and the nine engineered features provide minimal marginal gain (Δ*R*^2^ <0.001). This is not a contradiction but rather a reflection of the theoretical structure of basketball: win percentage is fundamentally determined by per-possession scoring differential (NRtg), whereas points per game depends on additional stylistic factors (pace, rebounding, scoring distribution) that the engineered features capture. The ablation results confirm that only OPP_FG_SUPP provides a small but detectable contribution (Δ*R*^2^ = +0.0003) beyond NRtg for WIN% modeling.

### 4.1 Methodological transferability

To empirically validate the transferability of the proposed framework beyond sports analytics, we replicated the entire pipeline, progressive feature sets, eight-model comparison, expanding-window validation, and systematic ablation, on a digital public health dataset: cross-country under-five mortality rate (U5MR, per 1,000 live births) estimation. The dataset comprises panel data from 15 countries spanning 1990–2020 (target variable: U5MR). Three hierarchical feature sets were constructed to mirror the WNBA design.

The specific feature counts at each tier (2, 5, 6) were constrained by publicly available data; however, the core principle of progressive enrichment, starting from parsimonious baselines and incrementally adding conceptually motivated features, remains methodologically identical across both domains. Set A [2 features: GDP per capita (log), Population (log)], Set B (5 features: Set A plus Health Expenditure %GDP, Primary School Enrollment, Basic Water Access), and Set C (6 features: Set B plus BCG Immunization Coverage). The expanding-window rolling validation covered 16 test years (2005–2020), and all eight models used identical hyperparameter grids as the WNBA experiments.

Table 13 presents the performance comparison across feature sets.

**Table 13:** Public health experiment: performance comparison across three feature sets (best model per feature set).

Feature set	Best model	*R* ^2^	RMSE	MAE
Set A (2 features)	RandomForest	0.724	18.11	8.69
Set B (5 features)	GradientBoosting	0.893	11.30	5.29
Set C (6 features)	GradientBoosting	0.922	9.65	5.01

The progressive feature engineering pattern observed in WNBA is replicated: adding socioeconomic and health system features (Set B) substantially improves estimation (Δ*R*^2^ = +0.169), and adding immunization coverage (Set C) provides a further meaningful improvement (Δ*R*^2^ = +0.029). Notably, the magnitude of improvement from Set B to Set C (Δ*R*^2^ = 0.029) is substantially larger than the corresponding improvement in the WNBA WIN% modeling (Δ*R*^2^ <0.001), suggesting that progressive feature engineering may be more impactful in public health contexts where additional features capture genuinely independent causal pathways.

Table 14 presents the eight-model comparison on Set C.

**Table 14:** Public health experiment: eight-model comparison on Set C (6 features).

Model	*R* ^2^	RMSE	Best Rank
GradientBoosting	**0.922**	**9.65**	1
XGBoost	0.911	10.31	2
RandomForest	0.884	11.76	3
ElasticNet	0.803	15.33	4
Ridge	0.803	15.33	4
Lasso	0.803	15.33	4
LinearRegression	0.803	15.33	4
SVR	0.302	28.84	8

The bold values indicate the best performance of each evaluation metric in this table.

A striking difference from the WNBA results is that Gradient Boosting, rather than a regularized linear model, achieves the best performance (*R*^2^ = 0.922). All four linear models cluster at *R*^2^ = 0.803, while tree-based ensemble methods achieve substantially higher *R*^2^ (0.884–0.922). This inversion is not a contradiction but rather evidence that the methodological framework, progressive feature sets, expanding-window validation, and systematic ablation, adapts to the data-generating process of the domain. In the WNBA, feature–target relationships are predominantly linear, favoring regularized linear models. In the public health data, nonlinear interactions among socioeconomic and health system variables (e.g., threshold effects of GDP on mortality reduction) favor ensemble tree methods. The framework correctly identifies the best model class in each domain without *a priori* assumptions.

Paired *t*-tests on the 16 fold-wise *R*^2^ values confirm that Gradient Boosting significantly outperforms Linear Regression (*t* = 2.417, *p* = 0.029, *), and SVR (*t* = 13.389, *p* < 0.001, ***), but the difference from RandomForest is not significant (*t* = 1.264, *p* = 0.226, ns). A bootstrap 95% confidence interval for the Gradient Boosting *R*^2^ is [0.761, 0.951].

For the closest tree-based competitors, Gradient Boosting vs. XGBoost showed no statistically significant difference (*t* = 0.821, *p* = 0.424, ns), consistent with the close *R*^2^ values (0.922 vs. 0.911). Nevertheless, Gradient Boosting consistently achieved the highest *R*^2^ across the 16 test folds and is therefore recommended as the preferred model for this task.

Table 15 presents the ablation experiment for Gradient Boosting on Set C.

**Table 15:** Public health experiment: ablation results for Gradient Boosting on Set C.

Feature removed	*R*^2^ (without)	*ΔR* ^2^	Contribution
Health_Expenditure_GDP	0.922	+0.0000	Positive (negligible)
Primary_School_Enrollment	0.911	+0.011	Positive
BCG_Immunization	0.893	+0.029	Positive
Basic_Water_Access	0.890	+0.032	Positive

The ablation results reveal that all four non-base features in Set C contribute positively to U5MR estimation. BCG Immunization Coverage and Basic Water Access show the largest marginal contributions (Δ*R*^2^ = +0.029 and +0.032, respectively), substantially larger than the corresponding ablation effects in the WNBA experiments. This confirms that progressive feature engineering is more impactful in public health, where added features often capture independent causal mechanisms (e.g., immunization programs reducing infectious disease mortality) rather than redundant stylistic information. The finding that BCG Immunization contributes a Δ*R*^2^ of 0.029, compared to the best WNBA engineered feature (REB_NET, Δ*R*^2^ = 0.0076), suggests that the marginal value of well-chosen features may be considerably higher in public health applications.

#### 4.1.1 Practical implications for U5MR modeling

For practitioners targeting U5MR estimation, these results offer clear prioritization guidance. First, we recommend prioritizing the collection of BCG immunization coverage and basic water access indicators, as these provide the largest marginal predictive gains (Δ*R*^2^≈0.029–0.032). In resource-limited settings, allocating data collection efforts toward these factors is likely to yield higher returns in model accuracy than investing in more granular economic metrics. Second, the marginal contribution of Health Expenditure (%GDP) is negligible (Δ*R*^2^≈0.0001), suggesting that this feature may be deprioritized when data acquisition is costly. Third, Gradient Boosting is the preferred algorithm for this task, outperforming all linear models by a substantial margin (Δ*R*^2^≈0.12), which implies that public health analysts should consider tree-based ensembles when modeling mortality outcomes with nonlinear socioeconomic determinants.

#### 4.1.2 Country-level sensitivity analysis

A country-level sensitivity analysis was also conducted. Linear models showed weak cross-country generalization, with negative *R*^2^ for most individual countries, indicating that a single linear specification cannot adequately capture the heterogeneity across nations. In contrast, Gradient Boosting maintained robust performance on the panel-level estimation task, demonstrating its ability to model country-level heterogeneity through nonlinear interactions. This finding mirrors the WNBA team-aware analysis, where small-sample subgroups showed weaker individual performance, and underscores a general principle: population-level estimation can be reliable even when individual-level (or country-level) models are underpowered.

Taken together, the public health transferability experiment demonstrates that: (1) the expanding-window validation framework and progressive feature engineering design are domain-agnostic and transportable; (2) the systematic ablation approach effectively identifies impactful features across domains; (3) the framework does not presuppose which model class will perform best, linear models excelled in WNBA while tree-based models excelled in public health, confirming its adaptability; and (4) the dual-value of the framework is validated: it serves both as a rigorous analytical tool within a domain and as a transferable methodology template across domains.

### 4.2 Broader methodological implications for digital public health

The public health transferability experiment (Section 4.1) provides concrete empirical evidence supporting the methodological framework's applicability beyond sports analytics. As noted by Albahli (12), temporal integrity is essential for reliable public health surveillance systems, and our expanding-window validation directly addresses this requirement. The U5MR experiment demonstrates that the framework achieves *R*^2^ = 0.922 with progressive feature engineering, from basic economic indicators (Set A, *R*^2^ = 0.724) to enriched health system features (Set C, *R*^2^ = 0.922), confirming that systematic feature set construction yields substantial gains in public health contexts.

Importantly, the fact that Gradient Boosting, rather than regularized linear regression, emerged as the best model for U5MR estimation, while regularized linear models dominated in the WNBA, is a strength rather than a limitation of the framework. This domain-dependent model selection confirms that the proposed methodology does not impose *a priori* assumptions about the appropriate model class; rather, it provides a rigorous comparative infrastructure that lets the data determine the best approach. For public health practitioners, this means the framework can be confidently applied to new domains without presupposing linear or nonlinear dominance.

The systematic ablation results further support the transferability of our “feature selection is as important as feature creation” principle. In the U5MR experiment, BCG Immunization Coverage contributed Δ*R*^2^ = +0.029, nearly four times the largest ablation effect in the WNBA (REB_NET, Δ*R*^2^ = +0.0076). This larger marginal contribution in public health reflects the fact that well-constructed features in this domain often capture independent causal mechanisms, whereas sports features frequently overlap with existing efficiency metrics. This observation has direct practical implications: public health researchers should invest in progressive feature engineering, as each additional well-designed feature is more likely to provide genuine incremental value.

The target-dependent feature utility observed in the WNBA (PPG requiring richer features than WIN%) mirrors the distinction in public health between *process indicators* (e.g., physical activity minutes, screening uptake) and *outcome indicators* (e.g., disease incidence, mortality). Just as net rating (NRtg) overwhelmingly determined win percentage while scoring required more nuanced features, public health outcome models may benefit from simpler, more robust predictors, whereas process models may require richer feature engineering (26).

For practitioners, we recommend: (1) For PPG modeling, use the optimized 12-feature set (Set B plus REB_NET, Q_VOL, and C_REB_RATE); expected error ≈ 1.2 points. (2) For WIN% modeling, prioritize accurate NRtg estimation; the full 18-feature set provides no meaningful advantage over Set B, and expected error ≈ 3.9 percentage points. (3) Always validate engineered features via ablation before deployment, as feature-target alignment determines whether additional complexity yields performance gains. (4) For public health applications adopting this framework, begin with parsimonious base feature sets and progressively enrich, using ablation to confirm each addition's marginal contribution rather than assuming that more features improve estimation.

### 4.3 Scope of the estimation framework: lagged boundary analysis

To delineate the scope of the proposed within-season estimation framework and to directly address whether it can be extended to forward prediction, we conducted a boundary analysis using lagged features. Specifically, we constructed a lagged dataset where the features from season *t*−1 were paired with the target variable from season *t* (i.e., using last-season statistics to estimate the current-season target, analogous to a forward-prediction scenario), and applied the same expanding-window rolling validation and Set C features under identical model configurations. Table 16 summarizes the results alongside the original within-season estimates.

**Table 16:** Within-season estimation vs. lagged prediction boundary analysis (Set C, expanding-window validation, 2011–2025).

Target	Model	Within-season *R*^2^	Lagged *R*^2^	*ΔR* ^2^
PPG	Lasso	0.9443	−0.045	−0.989
WIN%	ElasticNet	0.9287	0.163	−0.766

Lagged prediction uses *t*−1 season features to estimate *t* season targets. The substantial *R*^2^ decline confirms that the framework captures concurrent feature–target associations rather than forward-looking predictive power.

The lagged boundary analysis reveals a dramatic performance decline: under the same model configurations, PPG estimation accuracy drops from *R*^2^ = 0.9443 (within-season) to *R*^2^ = −0.045 (lagged), and WIN% drops from *R*^2^ = 0.9287 to *R*^2^ = 0.163 (Table 16). Across all eight models, no model achieved a lagged *R*^2^ above 0.17 for either target.

This dramatic decline is not unexpected and aligns with the inherently low year-to-year autocorrelation of WNBA team-level statistics, indicating that a team's statistical profile in one season is a weak predictor of its profile in the next. Beyond this statistical property, substantial roster turnover, coaching changes, and league-level evolution between seasons further sever the link between a team's statistics in one season and its outcomes in the next. The concurrent within-season features (e.g., shooting efficiency and rebounding in the same season) are strongly correlated with same-season outcomes because they share a common causal pathway: the team's actual on-court performance, whereas year-over-year persistence is weak.

This finding is important for three reasons. First, it transparently delineates the scope of our framework: the high *R*^2^ values reported in this study reflect within-season estimation accuracy, not forward prediction capability. We use the term “estimation” rather than “prediction” throughout this paper to maintain terminological precision. Second, the poor lagged performance does not undermine the framework's utility: it is precisely the absence of strong year-over-year autocorrelation that motivates within-season estimation as the appropriate analytical goal. Third, in the public health context, the situation may differ: many health indicators (e.g., GDP, immunization coverage) exhibit strong temporal persistence, and lagged prediction may perform more favorably in domains where structural factors evolve gradually. This contrast further highlights the domain-dependent nature of model applicability and reinforces the value of our expanding-window framework as a flexible infrastructure that can accommodate both concurrent estimation and lagged prediction depending on the data-generating process.

### 4.4 Limitations

Several limitations should be acknowledged. First, the WNBA sample size remains modest (242 team-season observations across 20 seasons and 12 teams), which limits the statistical power for detecting small effect sizes and may inflate *R*^2^ estimates through overfitting to the specific sample composition. Second, the multicollinearity among features, particularly the high correlations among efficiency metrics (TS%, FG%, NRtg, all with *r*>0.8), complicates the interpretation of individual regression coefficients, as the conditional associations captured by Lasso may not reflect independent causal effects. Third, multiple observations from the same team across seasons are not statistically independent; a team's performance in consecutive years is likely autocorrelated, which may lead to underestimated standard errors and overconfident significance tests. This intra-team dependence also limits the interpretability of team-aware sensitivity analysis for teams with very small sample sizes. Fourth, the public health transferability experiment has its own limitations: the country-level sensitivity analysis revealed that linear models generalize poorly across individual countries, and the panel-level *R*^2^ values are partly driven by between-country variation rather than within-country temporal dynamics. Further validation on additional public health datasets with larger cross-sectional samples is needed. Fifth, as the lagged boundary analysis (Section 4.3) confirms, this study employs a within-season estimation framework: models estimate current-season performance using current-season features, and forward prediction from lagged features alone yields substantially lower accuracy (*R*^2^ <0.17). This design is appropriate for quantifying team performance ex post and evaluating how well historical relationships generalize to unseen seasons, but it does not address prospective forecasting. Sixth, team-season aggregation necessarily misses game-level dynamics, and exclusion of off-court factors (injuries, coaching changes, roster turnover) limits the completeness of the feature space.

## 5 Conclusion

Regularized linear regression achieves high estimation accuracy for WNBA team performance under small-sample constraints. Lasso yields the highest *R*^2^ (0.9443) for PPG; ElasticNet yields the highest *R*^2^ (0.9287) for WIN%, though pairwise comparisons among the regularized linear models reveal no statistically significant differences for the closest competitors, indicating that the primary advantage lies in the linear family as a whole rather than in any single specification. The expanding-window validation framework ensures temporal integrity.

A key methodological contribution is the demonstration that **feature selection matters as much as feature creation, and the value of engineered features depends on the target variable**. For PPG, only 3 of 9 engineered features contribute positively, and the optimized 12-feature set outperforms the full 18-feature set (*R*^2^ = 0.9497 vs. 0.9443). For WIN%, the advanced metrics in Set B already capture most variance, with engineered features providing minimal marginal gain (Δ*R*^2^ <0.001), confirming that net rating overwhelmingly determines win percentage. This target-dependent feature utility highlights the importance of systematic ablation analysis to distinguish truly incremental features from redundant ones.

Beyond its contributions to sports analytics, this study offers a methodological template for digital public health research facing small-sample, temporally structured data. The expanding-window validation framework ensures temporal integrity; the systematic ablation approach distinguishes truly incremental features from redundant ones; and the finding that parsimonious models (12 features) can outperform full models (18 features) challenges the assumption that more data complexity always improves estimation (15, 26). The empirical validation on cross-country under-five mortality data (*R*^2^ = 0.922) further demonstrates that the framework adapts to domains with different data-generating processes, where nonlinear models may outperform linear ones. As digital public health increasingly adopts machine learning for population health surveillance and intervention evaluation, rigorous validation frameworks like the one proposed here will be essential for building reliable, generalizable estimation models.

Future work should extend to game-level estimation, cross-league transfer learning, real-time applications, and forward prediction using lagged features.

## Data Availability

The datasets presented in this study can be found in online repositories. The names of the repository/repositories and accession number(s) can be found in the article/Supplementary material.
